# The role of GZMA as a target of cysteine and biomarker in Alzheimer’s disease, pelvic organ prolapse, and tumor progression

**DOI:** 10.3389/fphar.2024.1447605

**Published:** 2024-08-20

**Authors:** Yan Li, Zhuo Wang, Min Kong, Yuanyuan Yong, Xin Yang, Chongdong Liu

**Affiliations:** ^1^ Department of Gynecology and Obstetrics, Affiliated Beijing Chaoyang Hospital of Capital Medical University, Beijing, China; ^2^ Department of Gynecology and Obstetrics, General Hospital of Ningxia Medical University, Yinchuan, China; ^3^ Department of Gynecology and Obstetrics, Ningxia Medical University, Yinchuan, China; ^4^ Department of Gynecology and Obstetrics, Chaoyang Hospital Affiliated to Capital Medical University, Beijing, China

**Keywords:** alzheimer’s disease, pelvic organ prolapse, multi-omics analysis, metabolomics, transcriptomics

## Abstract

**Objective:** This study aims to investigate how changes in peripheral blood metabolites in Alzheimer’s Disease (AD) patients affect the development of Pelvic Organ Prolapse (POP) using a multi-omics approach. We specifically explore the interactions of signaling pathways, gene expression, and protein-metabolite interactions, with a focus on GZMA and cysteine in age-related diseases.

**Methods:** This study utilized multi-omics analysis, including metabolomics and transcriptomics, to evaluate the perturbations in peripheral blood metabolites and their effect on POP in AD patients. Additionally, a comprehensive pan-cancer and immune infiltration analysis was performed on the core targets of AD combined with POP, exploring their potential roles in tumor progression and elucidating their pharmacological relevance to solid tumors.

**Results:** We identified 47 differential metabolites linked to 9 significant signaling pathways, such as unsaturated fatty acid biosynthesis and amino acid metabolism. A thorough gene expression analysis revealed numerous differentially expressed genes (DEGs), with Gene Set Enrichment Analysis (GSEA) showing significant changes in gene profiles of AD and POP. Network topology analysis highlighted central nodes in the AD-POP co-expressed genes network. Functional analyses indicated involvement in critical biological processes and pathways. Molecular docking studies showed strong interactions between cysteine and proteins PTGS2 and GZMA, and molecular dynamics simulations confirmed the stability of these complexes. *In vitro* validation demonstrated that cysteine reduced ROS levels and protected cell viability. GZMA was widely expressed in various cancers, associated with immune cells, and correlated with patient survival prognosis.

**Conclusion:** Multi-omics analysis revealed the role of peripheral blood metabolites in the molecular dynamics of AD and their interactions with POP. This study identified potential biomarkers and therapeutic targets, emphasizing the effectiveness of integrative approaches in treating AD and POP concurrently. The findings highlight the need for in-depth research on novel targets and biomarkers to advance therapeutic strategies.

## Highlights

Our study harnesses multi-omics to correlate peripheral blood metabolite variations with Pelvic Organ Prolapse (POP) in Alzheimer’s Disease (AD) patients, uncovering 47 metabolites across critical pathways. Notably, cysteine’s interaction with GZMA suggests novel AD treatment strategies, presenting a breakthrough in integrating traditional therapies with molecular medicine to potentially improve POP management in AD, promising for both early diagnosis and targeted treatment.
This aligns with the growing emphasis on exploring novel targets and biomarkers in advancing drug development and treatment modalities, particularly within the context of solid tumors.

## 1 Introduction

With the surge in healthcare demands, dementia, particularly Alzheimer’s disease (AD), has become a prevalent and progressive neurodegenerative condition, accounting for 60%–70% of global cases (Li et al., 2022). China is facing a rapid increase in the prevalence of AD, with predictions indicating that nearly 48.98 million people will be affected by 2050 (Li F. et al., 2021). This condition not only deteriorates human health and quality of life but also imposes a significant economic burden, with the cost of AD in Zhejiang Province alone reaching 27.53 billion RMB in 2017, accounting for 0.77% of its GDP (Yu et al., 2021). Similarly, pelvic organ prolapse (POP), another popular disease among the elderly, has drawn attention due to its rising incidence. The incidence rate in China is 9.6%, and it increases with age (Pang et al., 2021). It is estimated that the incidence rate in the United States will increase to 46% by 2050 (Weintraub et al., 2020).

The development of AD is closely linked to metabolic disturbances both centrally and peripherally. The exploration of clear metabolic markers for AD risk is ongoing, there is substantial evidence linking peripheral blood metabolites to AD phenotypes (Huo et al., 2020). For instance, dysregulation in peripheral phosphatidylcholine metabolism, which play a crucial role in early AD pathology, may lead to the accumulation of β-amyloid protein in the brain (Nho et al., 2021). Lys phosphatidylcholine (LPC), as a positively associated biomarker with neurodegenerative diseases (Law et al., 2019), along with other peripheral lipid metabolites, exerts a significant influence on the progression of mild cognitive impairment (MCI) to AD (Blasko et al., 2021). Additionally, peripheral metabolic alterations in AD can trigger additional pathological mechanisms, such as those mediated by insulin resistance, which is a condition linked to AD and metabolic diseases such as NAFLD (Le Stunff et al., 2019). The peripheral kynurenine pathway, involving the enzyme kynurenine (KYNs), has implications for cognitive impairment and is associated with a range of diseases (Gulaj et al., 2010; Török et al., 2020). The application of big data and bioinformatics in the identification and utilization of biomarkers plays an increasingly important role in disease diagnosis and prognosis evaluation. Through multi-omics analysis and biomarker research, scientists can more precisely identify and validate disease-related biomarkers (Gao et al., 2024; Xia et al., 2024). The development of new targeted therapy strategies promises to enhance treatment efficacy and reduce adverse reactions, thus promoting the advancement of precision medicine (Chen B. et al., 2024; Kang et al., 2024). These numerous research outcomes not only provide novel insights and methods within their respective fields but also demonstrate the enormous potential of interdisciplinary collaboration in disease diagnosis and treatment (Kong et al., 2024; Wahi et al., 2024). The open access and cross-disciplinary application of these studies further propel the development of precision medicine, emphasizing the importance of integrated data analysis and multi-dimensional assessment in modern medicine (Oinaka et al., 2024; Xia and Ma, 2024; Xie et al., 2024; Yin et al., 2024).

There is an inherent connection between POP and AD. Research has found a close correlation between POP and low estrogen levels. The decrease in estrogen levels leads to vasoconstriction of pelvic floor muscles and fascia tissues, resulting in reduced blood supply and thinning, leading to a decrease in pelvic floor tissue tension and increased susceptibility to POP. In addition, the occurrence of POP is closely related to oxidative stress. The significant increase in ROS levels suggests that mechanical force can cause an increase in ROS levels in pelvic floor support tissue, leading to oxidative stress and oxidative damage, ultimately resulting in POP. Meanwhile, estrogen, as a natural antioxidant, can inhibit neuronal degeneration caused by oxidative stress and alleviate mitochondrial damage caused by increased oxygen free radicals, thereby delaying neuronal aging and preventing the occurrence of AD. Plasma triglyceride and HDL cholesterol may have a role in maintaining BBB integrity in mild-to-moderate Alzheimer’s disease, which may affect the severity of the condition (Rogowski et al., 2015). Traditional research has focused on tissue markers, such as the relationship between MMP-1 expression and urogenital tract prolapse (Strinic et al., 2009). The therapeutic potential of natural products, including flavonoids, alkaloids, glycosides, saponins, and polyphenols, in AD has been documented in various studies (Du and Liu, 2024; Yao et al., 2024). However, the influence of peripheral metabolic disorders on the progression of POP, especially in AD patients, remains still poorly studied and further exploration of molecular interactions between these diseases is needed.

In recent years, developing novel targeted therapeutic strategies to enhance treatment efficacy and minimize adverse effects has become a crucial direction in precision medicine, including the exploration of natural compounds as potential therapeutic agents (Mazumder et al., 2018; Tayeb et al., 2024). For example, by studying the effects of exercise on transcriptional regulatory characteristics in patients with AD, researchers have identified several potential therapeutic pathways (Chen et al., 2022a; Huang J. et al., 2022; Chen Y. et al., 2024). Natural compounds have shown promise in treating AD through various mechanisms, including anti-amyloidogenic, antioxidant, and anti-inflammatory effects (Andrade et al., 2019; Lye et al., 2021). Clinical trials have evaluated several natural products for AD, such as docosahexaenoic acid and cerebrolysin, which demonstrated potential cognitive benefits (Mohamed Yusof and Mohd, 2024). Phenolic compounds like myricetin, rosmarinic acid, and curcumin have exhibited anti-amyloidogenic properties in both *in vitro* and *in vivo* models (Yamada et al., 2015). Multi-targeted designed ligands inspired by natural sources have also been developed and studied using computational methods (Iqubal et al., 2021). Despite promising preclinical results, more well-designed clinical trials are needed to establish the efficacy of natural compounds in AD treatment (Bui and Nguyen, 2017; Andrade et al., 2023). POP is a common condition affecting women, with various treatment options available. Surgical interventions like uterosacral ligament suspension and sacrospinous ligament fixation show similar long-term outcomes for apical prolapse (Jelovsek et al., 2018). While synthetic mesh repairs demonstrate better anatomical results, they do not significantly improve quality of life compared to native tissue repairs and may lead to complications (Withagen et al., 2011). Conservative treatments, such as pelvic floor muscle training (PFMT), have shown promise in managing POP symptoms and potentially reversing prolapse (Hagen et al., 2009; Brækken et al., 2010). Although natural compounds have been explored for various conditions, no specific clinical trials were reported for POP treatment in the provided papers. Further research is needed to evaluate long-term outcomes and optimize treatment strategies for POP. Natural compounds have shown promising potential in cancer prevention and treatment through various mechanisms. Clinical trials and animal studies have demonstrated the immunomodulatory effects of compounds like carotenoids, curcumin, resveratrol, EGCG, and β-glucans in inhibiting tumor progression (Pan et al., 2019). Terpenoids such as glycyrrhizic acid, ursolic acid, and limonene exhibit antitumor and anti-angiogenic properties (Kuttan et al., 2011). Plant-derived compounds like paclitaxel and homoharringtonine are already in clinical use, while others like curcumin and ingenol mebutate are in clinical trials (Seca and Pinto, 2018). Proteasome inhibitors, including bortezomib and natural products like EGCG and genistein, have shown efficacy in cancer treatment (Yang et al., 2009). The pipeline for natural product-derived drugs is promising, with numerous compounds in various stages of clinical trials, particularly for oncology and anti-infective applications (Butler, 2008). However, further evidence is needed to corroborate these findings. Overall, the interplay between AD, POP, and tumor improvement presents a complex clinical challenge that requires in-depth interdisciplinary research to discover.

Overall, the interplay between AD, POP, and tumor improvement presents a complex clinical challenge that requires in-depth interdisciplinary research to discover. Although current studies suggest organic links between these conditions, the specific mechanisms and influencing factors require additional investigation. The integration of multi-omics data with advanced bioinformatics analysis is anticipated to further advance precision medicine, providing a more scientific basis for disease diagnosis and treatment (Chen et al., 2019; Chen et al., 2022b; Lu et al., 2022).

## 2 Materials and methods

### 2.1 Metabolomic analysis of peripheral blood in AD patients

Peripheral blood metabolomic data from AD patients were obtained from the NCBI PubMed and GEO databases (http://www.ncbi.nlm.nih.gov/geo/). The UPLC-MS data were processed using Progenesis QI software for compound identification and multivariate statistical analysis. Principal Component Analysis (PCA) was applied to each dataset at various time points. Statistical tests were conducted to identify significant differences in ion content between the model and control groups, with ions exhibiting differences (*p* < 0.05) being considered as potential biomarkers. The tentative identification of these differential ions was achieved through the software’s compound identification function, utilizing retention time and mass-to-charge ratio data to query the HMDB metabolite database. A t-test was utilized to analyze statistical differences, and metabolic ions meeting the criteria of VIP>1 and *p* < 0.05 were deemed potential biomarkers. The identified differential metabolites underwent metabolic pathway enrichment analysis using the Pathway Analysis module in MetaboAnalyst 5.0. Additionally, a joint enrichment analysis was conducted in conjunction with AD transcriptome data to further clarify the implicated metabolic pathways.

### 2.2 Analysis of AD transcriptomics data

A search of the GEO database was executed using the terms “peripheral blood” and “AD,” limited to human studies. From the 6,278 and 528 series retrieved, the search was narrowed to include only non-interventional studies focusing on expression profiling of peripheral blood samples from AD patients or controls. Two datasets, GSE97760 and GSE168813, were ultimately selected for further analysis. The GSE97760 dataset comprised 9 peripheral blood samples from patients with advanced AD (average age 79.3 ± 12.3 years) and 10 samples from age-matched healthy female controls (average age 72.1 ± 13.1 years). The GSE168813 dataset included peripheral blood samples from 5 AD cases and 10 healthy controls (all female, average age 76.3 ± 3.5 years). The dataset GSE122063 includes gene expression profiling by array of 56 AD cases and 36 control samples, with samples taken from the frontal and temporal cortex. These samples, obtained from the University of Michigan Brain Bank, include non-demented controls and AD cases with no infarcts in the autopsied hemisphere. Differentially expressed genes (DEGs) between the AD and POP control groups were identified using the GEO2R online analysis tool, which facilitates the comparison of different sample groups within the GEO series to detect DEGs under various experimental conditions. The DEGs were selected based on the criteria of corrected *p* < 0.05 and |log2FC(fold change)| > 1.00. Volcano and heat maps of DEGs were created using R software (version 4.0.0). Venn diagrams were employed to identify common DEGs between the two datasets, indicative of AD-related DEGs. Further analysis involved constructing gene co-expression networks for the AD-related DEGs to explore the interrelationships among these genes.

### 2.3 Analysis of transcriptome data in POP

To analyze gene expression profiles related to POP, two datasets from the GEO database were identified: GSE12852 and GSE53868. These datasets encompassed transcriptome sequencing data and expression matrices from uterosacral ligament, round ligament tissue, and vaginal forearm tissue. The GSE12852 dataset, using the ABI Human Genome Survey Microarray Version 2, comprised 16 samples from uterosacral and round ligament tissues of POP patients and 18 samples from normal controls. The dataset GSE53868 includes 12 premenopausal women with POP, comparing tissues from prolapsed and non-prolapsed sites within the same patient. Whole genome GE 4 × 44 K microarrays were used to identify dysregulated pathways contributing to the pathogenesis of POP. The “edgeR” package in R was applied to identify DEGs between POP patients and healthy control tissues in both GSE12852 and GSE53868 datasets. Genes with |log_2_FC|>0.5 and *p* < 0.05 were classified as POP-associated DEGs. The analysis also included the generation of heat maps, volcano plots, and interaction relationship maps for the identified DEGs.

### 2.4 GSEA enrichment analysis

To conduct GSEA on the original dataset, the clusterProfiler package was employed. The gene sets C2.CP.KEGG.v7.4 and C5.GO.BP/CC/MF.v7.4 were obtained from the Molecular Characterization Database (http://www.gsea-msigdb.org/gsea/msigdb/index.jsp) for this particular investigation. The GSEA was conducted to detect enriched biological processes or pathways, by aggregating and removing duplicate leading-edge genes linked to these processes. The criteria used for screening enriched pathways included an absolute value of the corrected normalized enrichment score (NES) exceeding 1, a standardized significance level of *p* < 0.05, and a false discovery rate (FDR) below 0.25.

### 2.5 Identification of Co-Disease genes

To pinpoint genes associated with both AD and POP, the Venny 2.1.0 online tool was utilized to construct a Venn diagram of disease-related DEGs. The DEGs from the AD GSE97760 dataset were entered into the “List 1 column, while those from the AD GSE168813 dataset were entered into the “List 2 column. For POP, the DEGs from the GSE12852 dataset were input into the “List 3 column, and the DEGs from the GSE53868 dataset were placed into the “List 4 column. The Venn diagram generated from this process effectively delineated the intersecting genes, which were subsequently documented in the “Results” section as DEGs implicated in both AD and POP.

### 2.6 Construction of PPI network and identification of key genes

To map the protein-protein interaction (PPI) network, DEGs associated with both AD and POP were imported into the STRING 11.5 platform. The organism type was specified as “*Homo sapiens*,” and the minimum interaction threshold was set to 0.400. This configuration enabled the depiction of a PPI network that captures the interplay among AD + POP-related DEGs. Functional sub-clusters within this network were identified using the DMNC, Degree, and Closeness algorithms available in the “cytoHubba” plug-in of Cytoscape 3.7.2 software. The intersection of these algorithms’ results was used to determine the Hub genes, totaling (Chin et al., 2014). These Hub genes underwent enrichment analysis using the “ClueGO” plug-in in Cytoscape software.

### 2.7 Analysis of metabolic pathways and biological processes related to AD and POP

Genetic factors linked to AD and POP were examined through the utilization of the DAVID database (http://david.ncifcrf.gov/) for the purpose of conducting Gene Ontology (GO) enrichment and Kyoto Encyclopedia of Genes and Genomes (KEGG) pathway enrichment analyses. Findings meeting a significance threshold of *p* < 0.05 were graphically depicted to emphasize pertinent biological annotations.

### 2.8 Molecular docking

The 3D structures of the key differential metabolites found in the blood of AD patients were obtained from the PubChem database. Subsequently, genes from the most influential and central sub-clusters of the PPI network were selected for molecular docking studies. The 3D structures of the corresponding proteins of the core genes were retrieved from the Protein Data Bank (http://www.rcsb.org/) database. The core target protein was prepared using PyMol software to remove solvent molecules, etc., and then further hydrogenated and charged with AutoDockTools. The core target proteins and active compounds were saved as “pdbqt” format files, and the appropriate grid positions and sizes were set. Finally, the docking of components and targets was done by Autodock Vina. The clustering heat map was created in R, and the docking results were visualized by PyMol software to construct a molecular docking pattern map. Discovery Studio 2019 was utilized to identify docking sites and calculate the LibDockScore of flexible binding.

### 2.9 Molecular dynamics simulation

Molecular dynamics simulations were carried out through Discovery Studio 2019 software. Initially, the most stable model of the docked metabolite-protein complex was preserved, and the metabolites were transformed into molecular structure files in mol2 format. Proteins were also converted into molecular structure files, and topology files were created using Discovery Studio 2019. The system’s charge was neutralized employing the CHARMM36 force field and the Solvation module, which was supplemented with water molecules, sodium, or chloride ions, ensuring that atoms within the protein were at least 10 Å away from the water box’s boundary. The simulation temperature was established at 300 K. Before the simulation, the system underwent molecular mechanics optimization using 50,000 steps of the steepest descent method. The optimized systems were then equilibrated for the NVT and NPT ensembles with a step size of 2 fs for a total duration of 100 ps each, with system positions restricted during equilibration. Subsequent molecular dynamics simulations were conducted at 300 K for 100 ns with a simulation time step of 1 ns? Following the removal of periodic boundaries, the final structures of the generated trajectories were extracted at 20 ns intervals using the integrated tools of Discovery Studio 2019. These structures were aligned with the initial complexes to assess the interactions between the proteins and small molecules. The root mean square deviation (RMSD), root mean square fluctuation (RMSF), and hydrogen bonding heat maps of the protein-small molecule complexes were analyzed, and the data were imported into Prism for visualization.

### 2.10 GZMA pan-cancer expression landscape

In our study, we utilized the Wilcoxon rank-sum test to conduct a comparative analysis of gene expression levels between tumor and normal tissue specimens. The dataset was obtained from the TCGA project and standardized through the PanCanAtlas database. Specifically, we employed the EBPlusPlusAdjustPANCANIlluminaHiSeqRNASeqV2.geneExp.tv dataset, produced by the Firehose analysis pipeline, which integrates the MapSplice and RSEM algorithms. To enhance comparability, raw data underwent normalization by setting the upper quartile to 1,000, followed by Z-Score conversion to generate dimensionless standardized scores. Moreover, our study incorporated data from the HPA and GTEx projects to establish an RNA consensus tissue gene expression repository, encompassing 50 distinct tissue categories and qualified in nTPM values. For complex multi-sub-tissue structures such as the brain, lymph nodes, and intestines, the highest expression value from each sub-tissue was selected as the representative value. This repository, based on HPA version 23.0 and Ensembl version 109, amalgamated protein localization data from immunofluorescence staining and was formatted in a tab-delimited structure. The format included gene identifiers, names, reliability scores, location details, cell cycle dependencies, and GO cellular component term identifiers. Throughout our analysis, meticulous efforts were made to cleanse records with missing values to ensure the accuracy of the outcomes. We scrutinized 31 datasets from the HPA database, covering gene expression in 81 cell types, with a specific emphasis on 18 cell types and PBMC expression profiles. Additionally, we conducted a thorough assessment of gene expression trends across 28 cancer types to pinpoint gene expression alternations linked to cancer progression.

### 2.11 Clinical prognostic significance of GZMA in pan-cancer

This study aims to identify differences in gene expression between tumor and normal tissues. Primary datasets from the United States of America Jena database were utilized: CGAREMgeneTPM, which documents the TPM expression levels of tumor samples from the CGA project, and GTEREMgeneTPM, which captures the TPM expression levels of normal samples from the GTE project. Were subjected to Z-Score normalization applied to these datasets to mitigate the impact of differing scales and enhance comparability. During preprocessing, outliers with absolute Z-Score values exceeding 3.0 were excluded to minimize their potential influence on the analysis. To assess the significance of gene expression variances between tumor and normal tissues, the Wilcoxon rank-sum test, a non-parametric statistical method suitable for non-normally distributed data, was employed. Additionally, ROC analysis was conducted using the pROC package to assess the diagnostic potential of specific gene expression levels. This analysis involved determining the 95% confidence interval, AUC values, and plotting ROC curves to ascertain their efficacy in distinguishing between tumor and normal tissues. Our study also referred to the research by Thompson et al. (2018), which identified six molecular immune subtypes based on tumor molecular characteristics and patient prognosis. Tumor samples were stratified into high and low expression groups based on the median gene expression value. The distribution proportions of these groups across different molecular subtypes were analyzed using the Chi-square test to detect statistical variances. Specifically, the Kruskal–Wallis rank-sum test was applied to the BRA dataset to assess variations in GZMA gene expression among various molecular immune subtypes.

### 2.12 GZMA survival analysis

In this study, we conducted a comprehensive evaluation of the impact of gene expression levels on patient survival through Kaplan-Meier survival analysis. The R survival package was utilized for a thorough examination of survival data. The survminer package was employed to identify optimal cut-off values for various expression level groups, ensuring a balanced distribution of sample size, with each group representing at least 30% of the total sample size to enhance the statistical robustness of the analysis. We performed log-rank tests on various survival metrics, including Overall Survival (OS), Disease-Specific Survival (DSS), Progression-Free Survival (PFS), Progression-Free Interval (PFI), Disease-Free Survival (DFS), and Disease-Free Interval (DFI), to assess the impact of different expression levels on survival outcomes. Furthermore, we conducted a meta-analysis of univariate Cox proportional hazards models using the inverse variance method to integrate results from multiple studies. The primary measure of effect was the hazard ratio (HR), categorized into groups: HR < 1 (indicating potential tumor-suppressive effects) and HR > 1 (indicating potential tumor-promoting effects). While this classification may oversimplify the intricate relationship between gene expression and biological mechanisms, it offers a structured framework for analysis. For statistical analysis and visualization, we used the Meta package in R version 4.3.2, enabling the creation of forest plots and funnel plots to present combined effect sizes and assess potential publication bias.

### 2.13 GZMA single-gene GSVA enrichment analysis

In this study, a stratified method was employed to identify significant variances in gene expression. The top 30% of the sample distribution was categorized as the high-expression group, while the bottom 30% was classified as the low-expression group. This methodology was designed to emphasize noteworthy alternations in gene expression in the context of disease conditions and to investigate their biological implications. We utilized the Limma package in R, a widely recognized tool for differential expression analysis, to calculate the log2 fold changes (log_2_FC) of genes and to identify genes with significant expression changes by ranking them. Furthermore, we utilized the z-score algorithm with the GSA package in R to analyze 14 functional state gene sets. This transformation of gene set expression values into z-scores enabled the assessment of biological pathway activities. To further investigate the relationship between gene expression and functional states, we conducted Pearson correlation analysis to assess the statistical correlation between gene expression levels and gene set z-scores. By utilizing the GSA function in the GSA package, we scored 73 metabolic gene sets from the KEGG database and compared pathway activities between the high and low-expression groups using the Limma package. This comparison revealed the roles of these pathways in disease progression. For the examination of clinical variables, we divided patients into high and low-expression groups based on the median gene expression value, with the median serving as the cutoff for age grouping as well. Chi-square tests were conducted to identify differences in the distribution of various clinical variables between the two groups. This methodology helps identify associations between gene expression and clinical features, offering novel insights into the comprehension of disease mechanisms.

### 2.14 GZMA immune infiltration analysis

In this study, immune infiltration data were obtained from the TIMER 2.0 database, which integrates multiple algorithms to analyze the composition of immune cells in the tumor microenvironment and their relationship with gene expression. This integration ensures the accuracy and consistency of the data. The comprehensive analysis enabled us to explore the association between immune cells and gene expression levels. We used bar scatter plots to visualize the correlation coefficients, effectively demonstrating the interactions between various immune cell types and gene expression levels. To assess the correlation between transcription factor expression and ATAC peaks, with a focus on peaks located within 3,000 base pairs upstream and downstream of the target gene promoter regions, we employed the Spearman rank correlation coefficient, a non-parametric method. We calculated the correlation for each transcription factor and all peaks, with emphasis on results showing significant correlations (*p* < 0.01, cor>0). Furthermore, we retrieved RPPA protein expression data from the TCPA database and calculated activity scores for 10 cancer-related pathways based on literature references. By utilizing the cor.test function in R, we computed the Spearman correlation and *p*-values between the target gene and these pathway activity scores, further exploring the potential connections between gene expression and pathway activity. These findings offer novel insights into the role of gene expression within the tumor immune microenvironment.

### 2.15 Cell culture and transfection

C2C12 myoblasts, obtained from the Chinese Academy of Sciences in Shanghai, were cultured in a humidified incubator with 5% CO_2_ at 37°C, in high-glucose DMEM supplemented with 10% fetal bovine serum and 1% penicillin/streptomycin. For subsequent experiments, 2×10^6^ cells were seeded in 6-well plates. Transfections were carried out following the Lipofectamine 3,000 reagent protocol. When cells reached approximately 50% confluency, C2C12 or 293T cells were seeded and transduced with lentiviral vectors at appropriate titers. The study involved four experimental groups: the normal control (NC), the lentivirus-coated empty vector (EV), and the lentivirus-coated GZMA overexpression vector (OE-GZMA). Post 72 h of transfection, cells were harvested, total RNA was extracted, cDNA was synthesized, and the efficiency of GZMA transfection was quantified.

### 2.16 Real-time quantitative polymerase chain reaction (qPCR)

Total RNA was meticulously isolated employing the Trizol reagent. DNA purity was appraised via NanoDrop technology. qPCR primers for mRNA were furnished by RiboBio and synthesized by Sangon, Shanghai. TB Green™ Premix Ex Taq™ II was utilized for mRNA quantification. Relative expression was normalized to the NC group, calculated using the comparative CT method (2^−ΔΔCt^), and the assays were performed sextuply.

### 2.17 Western blot

Cell lysates were prepared using RIPA buffer supplemented with phenylmethanesulfonyl fluoride. Protein concentrations were measured with the BCA protein assay. Proteins were separated on a 10% SDS-PAGE gel and transferred to nitrocellulose membranes. The membranes were then blocked with 5% skim milk in PBST for 1 h. Primary antibodies were incubated with the membranes overnight at 4°C, followed by incubation with horseradish peroxidase-conjugated secondary antibodies at room temperature for 1 h at a 1:2000 dilution. Protein bands were detected, captured, and analyzed using the Syngene imaging system and ImageJ software.

### 2.18 CCK-8 assay

The proliferation of C2C12 myoblasts under different conditions (NC, EV, and OE-GZMA) was evaluated using the CCK-8 assay. Cells were seeded in 96-well plates and exposed to the respective treatments for 24 h. Subsequently, 10 µL of CCK-8 solution was added to each well, and the plates were incubated for 2 h. Absorbance was measured with a microplate reader, and cell viability was determined by comparing the mean absorbance ratios of the treated groups to the control group.

### 2.19 Immunofluorescence detection of GZMA expression

Immunofluorescence staining was employed to validate the expression of the GZMA protein in fibroblasts across different experimental groups. Cells, cultured on chamber slides to a 50% confluent were fixed with 4% paraformaldehyde. Following permeabilization with 0.4% Triton X-100, they were incubated with various primary antibodies overnight at 37°C. Nuclei were visualized using Hoechst 33,342 staining and examined under an Olympus BX72 fluorescence microscope, which was equipped with a DP51 camera (Olympus Optical Co., Ltd., Tokyo, Japan).

### 2.20 Statistical analysis

All experiments were conducted at least three times. Data were analyzed using GraphPad Prism 7.0. The Student’s t-test was utilized for comparisons between the two groups. A one-way ANOVA was applied to identify differences among multiple groups for both normal and non-normal distributions. A *p*-value< 0.05 was considered statistically significant.

## 3 Results

### 3.1 dentification of Differential Blood Metabolites in Alzheimer’s Disease.

**FIGURE 1:**
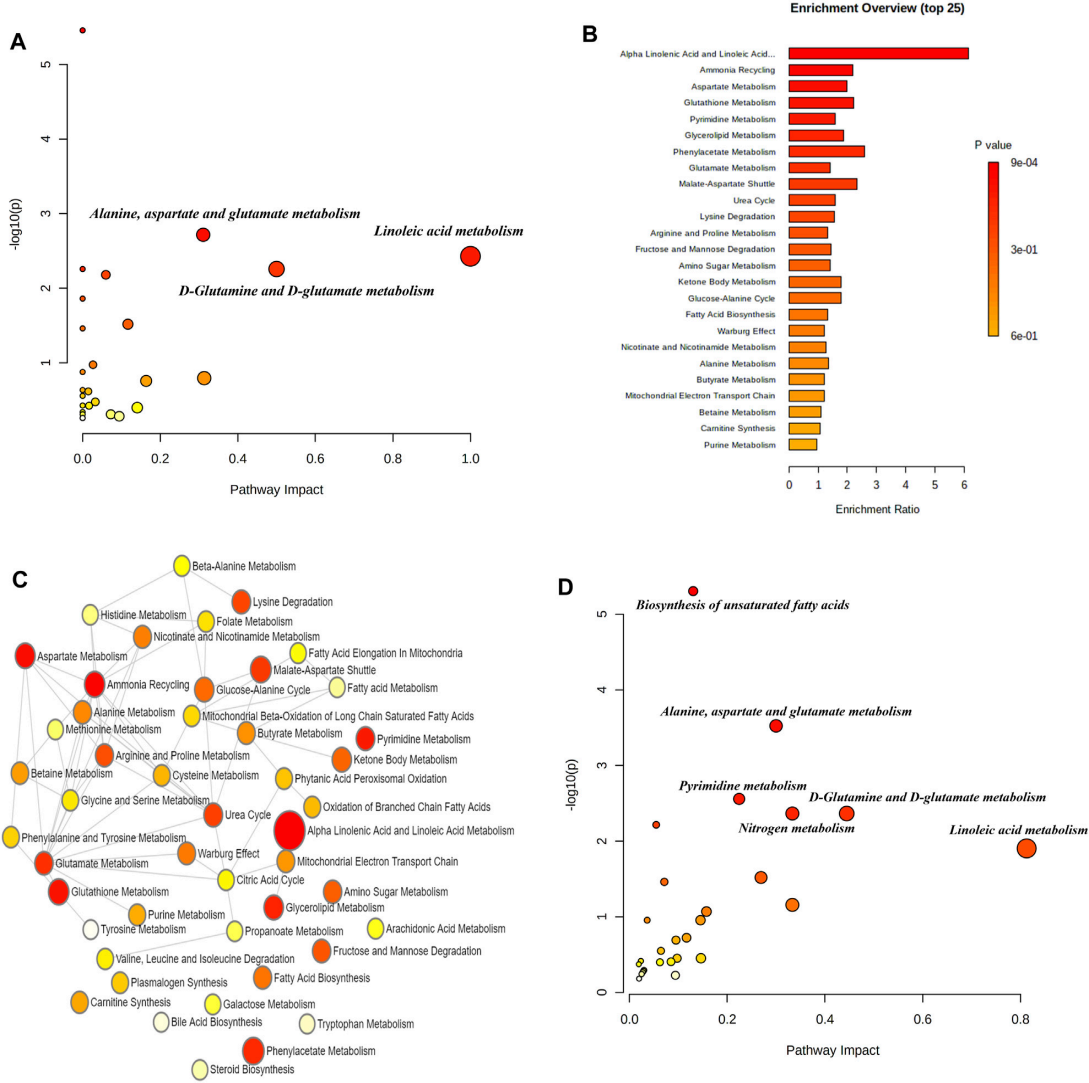
Metabolomic analysis of Alzheimer’s Disease (AD) peripheral blood. **(A)** Scatter plot showing the enrichment distribution of differentially expressed metabolite signaling pathways in AD peripheral blood. Key metabolic pathways are highlighted, such as alanine, aspartate and glutamate metabolism, and linoleic acid metabolism, with pathway impact on the x-axis and -log (*p*-value) on the y-axis, indicating the statistical significance of each pathway. **(B)** Histogram presenting the top 25 differentially expressed metabolite enrichment pathways in AD, sorted by enrichment ratio. Each bar is color-coded to reflect the *p*-value significance, with red indicating the most significant pathways. **(C)** Network diagram illustrating the complex interactions between different metabolic pathways affected in AD, with circles representing individual pathways and their connections indicating inter-pathway relationships. The size of each circle reflects the pathway impact, and the thickness of the connecting lines indicates the strength of the interaction. **(D)** Bubble chart representing the correlation between differentially expressed metabolites and differential gene enrichment in AD.

We identified a cohort of differential metabolites present in the blood samples of AD patients. Our study identified 47 distinct differential metabolites, encompassing significant biomarkers such as arachidonic acid, docosahexaenoic acid, linoleic acid, adrenic acid, elaidic acid, and palmitic acid. Subsequent metabolic pathway analysis conducted through the MetaboAnalyst 5.0 online database uncovered a total of nine signaling pathways significantly associated with these metabolites (*p* < 0.05). Notably, pathways involving the biosynthesis of unsaturated fatty acids, alanine, aspartate and glutamate metabolism, linoleic acid metabolism, and nitrogen metabolism were identified (Figure 1A). The differential metabolites showed substantial enrichment, particularly within the alpha-linolenic acid and linoleic acid metabolism pathways, as depicted in Figures 1B,C. Additionally, a comprehensive multi-omics enrichment analysis, integrating both differential blood metabolites and related differentially expressed genes in AD, highlighted predominant enrichment in several biological processes. These processes included the biosynthesis of unsaturated fatty acids, alanine, aspartate and glutamate metabolism, pyrimidine metabolism, nitrogen metabolism, D-glutamine and D-glutamate metabolism, aminoacyl-tRNA biosynthesis, linoleic acid metabolism, arginine biosynthesis, butanoate metabolism, among others (Figure 1D). The detection of these metabolites not only deepens our comprehension of AD-associated metabolic disruptions but also paves the way for potential biomarker development and therapeutic interventions.

### 3.2 Screening of AD-Related DEGs

**FIGURE 2:**
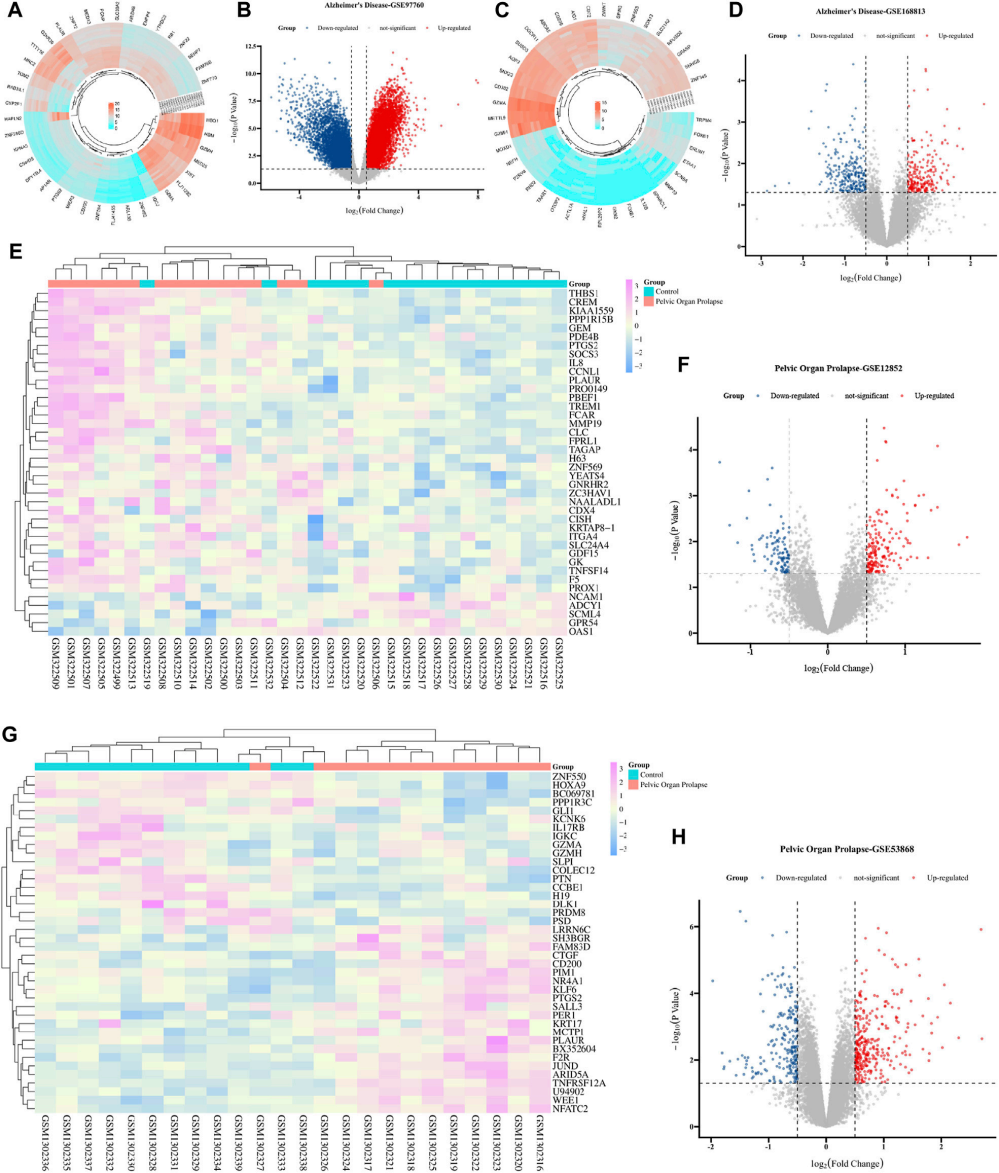
Comparative analysis of AD-related DEGs and POP-related DEGs across multiple studies. **(A)** Heat map of DEGs from the AD cohort in GSE97760, displaying the expression profiles across samples with colors indicating expression levels (red for high, blue for low). **(B)** Volcano plot for the same AD cohort in GSE97760, showing log2 (fold change) on the x-axis and -log10 (*p*-value) on the y-axis, with red dots indicating significantly upregulated genes and blue dots indicating significantly downregulated genes. **(C)** Heat map of DEGs from the cohort in GSE168813, with color intensity representing gene expression levels. **(D)** Volcano plot for the cohort in GSE168813, formatted similarly to **(B)**, with red and blue dots denoting up-and downregulated genes, respectively. **(E)** Heat map of DEGs in GSE12852, another AD cohort, with hierarchical clustering shown above the map. **(F)** Volcano plot of DEGs in GSE12852, with pink dots representing significantly upregulated genes and blue dots for downregulated genes. **(G)** Heat map of DEGs in GSE53868, displaying the clustering of gene expression patterns in a cohort. **(H)** Volcano plot for GSE53868, using the same color coding as **(F)**, to identify genes with significant changes in expression.

In the comprehensive screening of AD peripheral blood datasets, a considerable number of DEGs were identified. Within the GSE97760 dataset, we identified 7,370 DEGs, of which 4,003 were upregulated and 3,367 were downregulated. The clustering heat map and volcano map of the top 50 DEGs, selected based on the lowest *p*-value, revealed distinct gene expression profiles (Figures 2A,B). Similarly, the GSE168813 dataset yielded 499 DEGs, with 236 upregulated and 263 downregulated, and their expression patterns are illustrated in Figures 2C,D. The co-expression heat map of the top 50 most significant differential genes from these two AD datasets is presented in Supplementary Figures S1A & S1B. Further, the GSE12852 dataset, focusing on POP in uterosacral and round ligament tissues, revealed 282 DEGs, with 179 upregulated and 103 downregulated genes. The clustered heat map and volcano map of the top 50 DEGs are shown in Figures 2E,F. Lastly, the GSE53868 dataset analysis of anterior vaginal wall tissues from POP patients resulted in 539 DEGs, with 326 upregulated and 213 downregulated. Their expression profiles are depicted in Figures 2G,H, and the expression heat map of the most significant DEGs in both POP datasets (Supplementary Figure S1C, D).

### 3.3 GSEA functional enrichment analysis

GSEA of the AD dataset GSE97760 highlighted significant enrichment in several key gene sets, including HALLMARK E2F TARGETS, HALLMARK HEME METABOLISM, and others, indicating a strong association with AD pathology (Supplementary Figure S2A). The GSE168813 dataset analysis also revealed enrichment in AD-related gene sets, particularly those associated with cell cycle and inflammatory response (Supplementary Figure S2B). In the context of POP, the GSE12852 dataset showed a significant association with inflammatory and immune response-related gene sets (Supplementary Figure S2C), while the GSE53868 dataset indicated enrichment in gene sets related to inflammatory response and hypoxia (Supplementary Figure S2D). These enrichments propose a profound alteration within the gene expression panorama related to each AD and POP situation. The effects from the DEG screening and GSEA offer an in-depth and complete panorama of the gene expression changes in AD and POP.

### 3.4 Integrated analysis of DEGs in AD and POP

**FIGURE 3:**
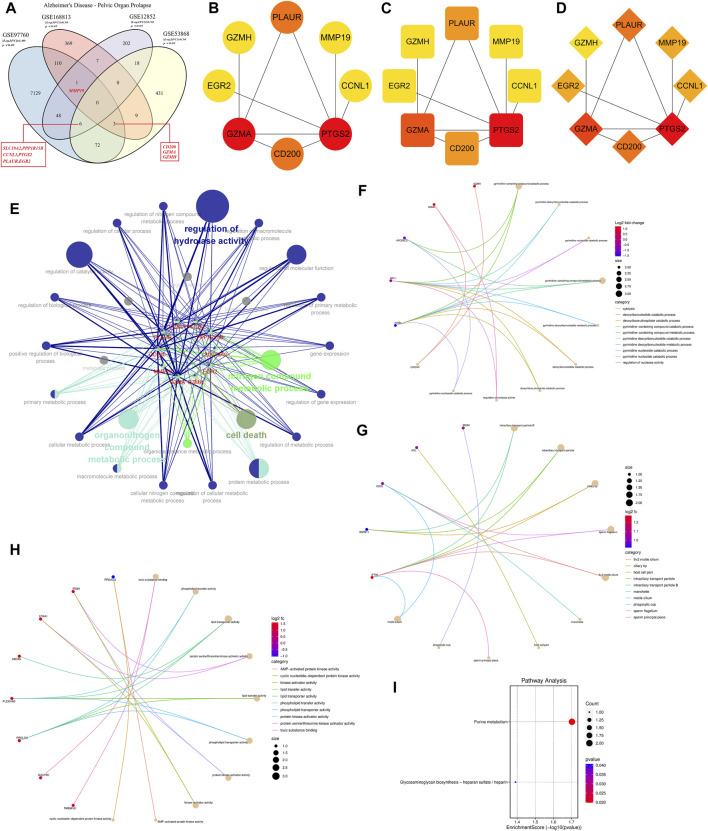
Protein-Protein Interaction (PPI) network construction and enrichment analysis of 43 Alzheimer’s Disease (AD)-related differentially expressed genes (DEGs). **(A)** Venn diagram showing the intersection of common targets among different disease states, facilitating the identification of potential biomarkers or therapeutic targets for AD. **(B)** Identification of potential core target genes in AD based on the Density of Maximum Neighborhood Component (DMNC) value, with the color intensity corresponding to the magnitude of the DMNC value. **(C)** Potential core target genes are ranked by Degree value, which is indicative of the number of connections a gene has within the PPI network, with darker colors representing higher values. **(D)** Potential core target genes are sorted by Closeness value, a measure of how close a gene is to all other genes in the network, with the color gradient representing the value magnitude. **(E)** Enrichment analysis network depicting the core disease targets and their associations with biological processes (BP), cellular components (CC), and molecular functions (MF). Node size and color intensity indicate the significance and the number of connections, respectively. **(F)** Chord diagram illustrating the BP functional analysis of the AD-related DEGs, with ribbons connecting genes to their associated biological processes. **(G)** Chord diagram for CC functional analysis, displaying the association between genes and the cellular components where they are active. **(H)** Chord diagram for MF functional analysis, showing the involvement of genes in specific molecular functions. **(I)** Bubble diagram of the Kyoto Encyclopedia of Genes and Genomes (KEGG) pathway analysis, with the size of each bubble corresponding to the gene count involved in the pathway, and the color indicating the enrichment significance. Pathways are ranked by *p*-value, with darker colors denoting higher significance.

Using an integrative approach to uncover commonalities between AD and POP, we analyzed datasets GSE97760, GSE168813, and GSE12852 with the “Venn Diagram” package in R This evaluation pinpointed MMP19 as a shared DEG across the initial datasets. Expanding the intersection to encompass GSE12852 and GSE53868 datasets yielded six genes (SLC19A2, PPP1R15B, CCNL1, PTGS2, PLAUR, and EGR2). Furthermore, a 3-manner intersection of the GSE97760, GSE168813, and GSE53868 datasets revealed an overlap of 3 genes (CD200, GMA, and GZMH) (Figure 3A). Subsequently, these intersecting genes were mapped onto a STRING database-derived PPI network of AD-POP co-expressed genes. Through network topology analysis using three distinct computational methods, we identified six key genes—PTGS2, GZMA, PLAUR, CD200, GZMH, and MMP19—as central nodes within this network (Figures 3B–D). Functional enrichment analysis, conducted with the “ClueGO” tool, indicated a significant association of these genes with biological processes such as hydrolase activity regulation, nitrogen compound metabolism, and cell death (Figure 3E). These findings underscore a subset of genes that may be fundamental to the shared pathological features between AD and POP, suggesting a convergence of molecular mechanisms that could lead to novel therapeutic insights.

### 3.5 Metabolic pathway and biological process analysis of key targets in AD and POP

A thorough investigation into the biological significance of AD-related core targets was conducted by importing the Uni Prot IDs of 43 crucial targets into the DAVID database for GO enrichment analysis. This extensive analysis delineated that the AD-related targets were predominantly involved in a myriad of biological processes, with a total of 149 being identified. Among these, the most significant processes associated with the peripheral blood marker genes of AD included the pyrimidine-containing compound catabolic process, deoxyribonucleotide catabolic process, and other pyrimidine-related metabolic pathways (Figures 3F–H). Additionally, KEGG database enrichment analysis of core targets revealed two pathways significantly enriched by these core targets, namely, Purine metabolism and Glycosaminoglycan biosynthesis - heparan sulfate/heparin (Figure 3I).

**FIGURE 4:**
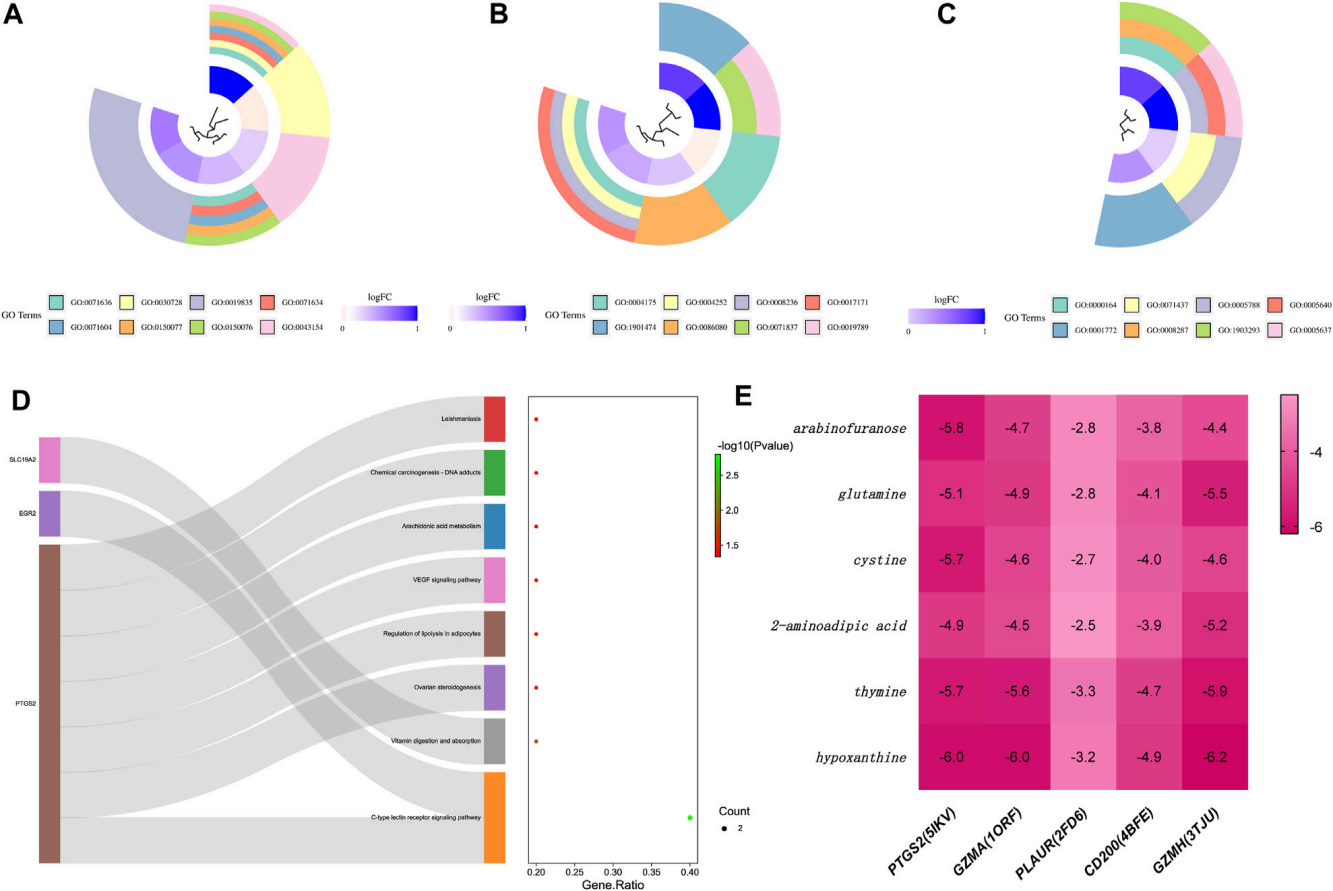
Functional and pathway enrichment analysis of common target genes associated with disease, and interaction analysis of core proteins with marker metabolites. **(A)** Loop diagram representing the Biological Process (BP) functional analysis of common target genes. Each segment of the loop corresponds to a different BP category, with colors and loop lengths representing the number of genes associated with each process. **(B)** Loop diagram of Cellular Component (CC) functional analysis, structured similarly to the BP loop diagram, detailing the cellular components associated with the common target genes. **(C)** Loop diagram illustrating the Molecular Function (MF) functional analysis, again using segments to denote various molecular functions attributed to the target genes. **(D)** Sankey diagram displaying the Kyoto Encyclopedia of Genes and Genomes (KEGG) pathway analysis. The diagram connects the target genes on the left to their corresponding pathways on the right, with the width of the connecting bands proportional to the gene count in each pathway. **(E)** Heat map of the binding energy between core proteins and marker metabolites, with the intensity of the color representing the strength of the binding affinity. Darker colors indicate a firmer binding between the protein and metabolite, suggestive of a stronger interaction. Each row represents a core protein, while each column corresponds to a specific marker metabolite.

Complementing this, GO enrichment analysis of the ten common DEGs obtained previously showed that the enriched biological processes (BP) primarily comprised positive regulation of transforming growth factor beta production and other significant processes like ovulation and cytolysis (Figure 4A). The molecular function (MF) category was dominated by activities related to endopeptidase, serine-type peptidase, and protein binding involved in heterotypic cell-cell adhesion (Figure 4B). Cellular components (CC) such as the protein phosphatase type 1 complex and immunological synapse were also prominently enriched (Figure 4C). The KEGG pathways that were significantly represented included the C-type lectin receptor signaling pathway and arachidonic acid metabolism, among others, suggesting a broad range of affected pathways (Figure 4D).

### 3.6 Molecular docking results of metabolic markers to core proteins

Molecular docking studies of six metabolic markers, which showed the most significant differential expression among five core proteins, were meticulously performed using the AutoDock-Vina program and Discovery Studio 2019 software. The primary screening heat map of binding energies indicated that arabinofuranose, glutamine, cysteine, thymine, and hypoxanthine all successfully complexed with the protein PTGS2. These complexes showed binding energies below −5.0 kcal/mol, RMSD values less than 2.00, and positive LibDockScores (Figure 4E). Notably, thymine and hypoxanthine were able to establish complexes with the protein GZMA, adhering to the same rigorous binding criteria. However, none of the metabolic markers could achieve favorable docking with the protein PLAUR, as evidenced by binding energies that did not exceed the threshold of −5.0 kcal/mol. Similarly, the protein CD200 did not form an effective docking with the metabolic markers. In contrast, protein GZMH demonstrated substantial docking potential with metabolic markers glutamine, 2-aminoadipic acid, thymine, and hypoxanthine. This was evidenced by binding energies below −5.0 kcal/mol and RMSD values that fell within the desired range.

**FIGURE 5:**
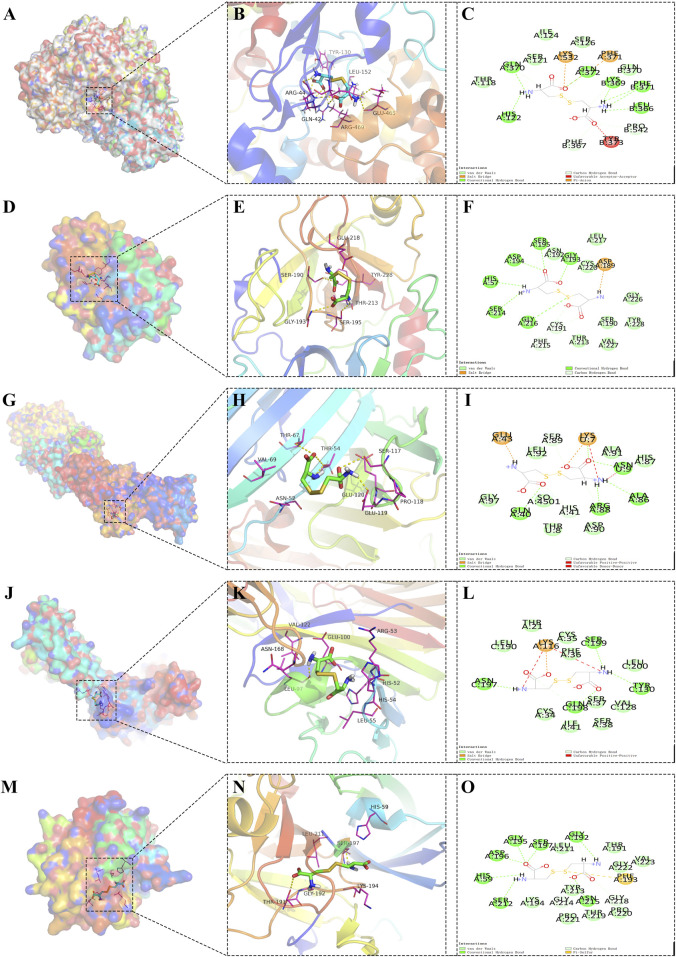
Docking models of core protein-metabolite complexes, illustrating the interactions between cysteine and various proteins implicated in disease processes. **(A)** Three-dimensional (3D) structure of the PTGS2 protein bound with cysteine, providing an overall view of the molecular docking arrangement. **(B)** Detailed local view of the 3D docking model showing the specific binding site and interactions of PTGS2 with cysteine. **(C)** Two-dimensional (2D) interaction diagram of PTGS2-cysteine, highlighting the amino acids involved in the binding and their relative positions. **(D)** 3D representation of the GZMA protein in complex with cysteine, showing the overall structure. **(E)** Zoomed-in 3D view of the GZMA-cysteine binding site, depicting the interaction points and the molecular conformation at the binding site. **(F)** 2D schematic of the GZMA-cysteine interaction, illustrating the points of contact between the amino acids of GZMA and cysteine. **(G)** 3D global structure of the PLAUR protein complexed with cysteine. **(H)** Close-up 3D view of the PLAUR-cysteine interaction, focusing on the binding pocket and the involved residues. **(I)** 2D diagram of the PLAUR-cysteine complex, showing the specific interactions and the spatial arrangement of the binding residues. **(J)** Overall 3D structure of the CD200 protein docked with cysteine. **(K)** Detailed 3D view of the CD200-cysteine complex at the binding site, with an emphasis on the interaction interface. **(L)** 2D representation of the CD200-cysteine interactions, detailing the binding residues and their interactions with cysteine. **(M)** 3D global visualization of the GZMH protein bound with cysteine. **(N)** Localized 3D view of the GZMH-cysteine binding site, showing the molecular interactions and conformation of the complex. **(O)** 2D interaction map of GZMH-cysteine, outlining the amino acids of GZMH involved in the binding and their spatial configuration.

Synthesizing these findings, it was observed that the core protein GZMA formed the most stable docking model with the metabolite cysteine, closely followed by GZMH with the same metabolite, implying a robust interaction (Figure 5). These interactions suggest that targeting the binding affinity of GZMA to cysteine, potentially by inhibiting its active center, may offer therapeutic benefits for AD and POP, providing a promising avenue for the development of novel treatment strategies.

### 3.7 Molecular dynamics simulations reveal stable protein-metabolite interactions

**FIGURE 6:**
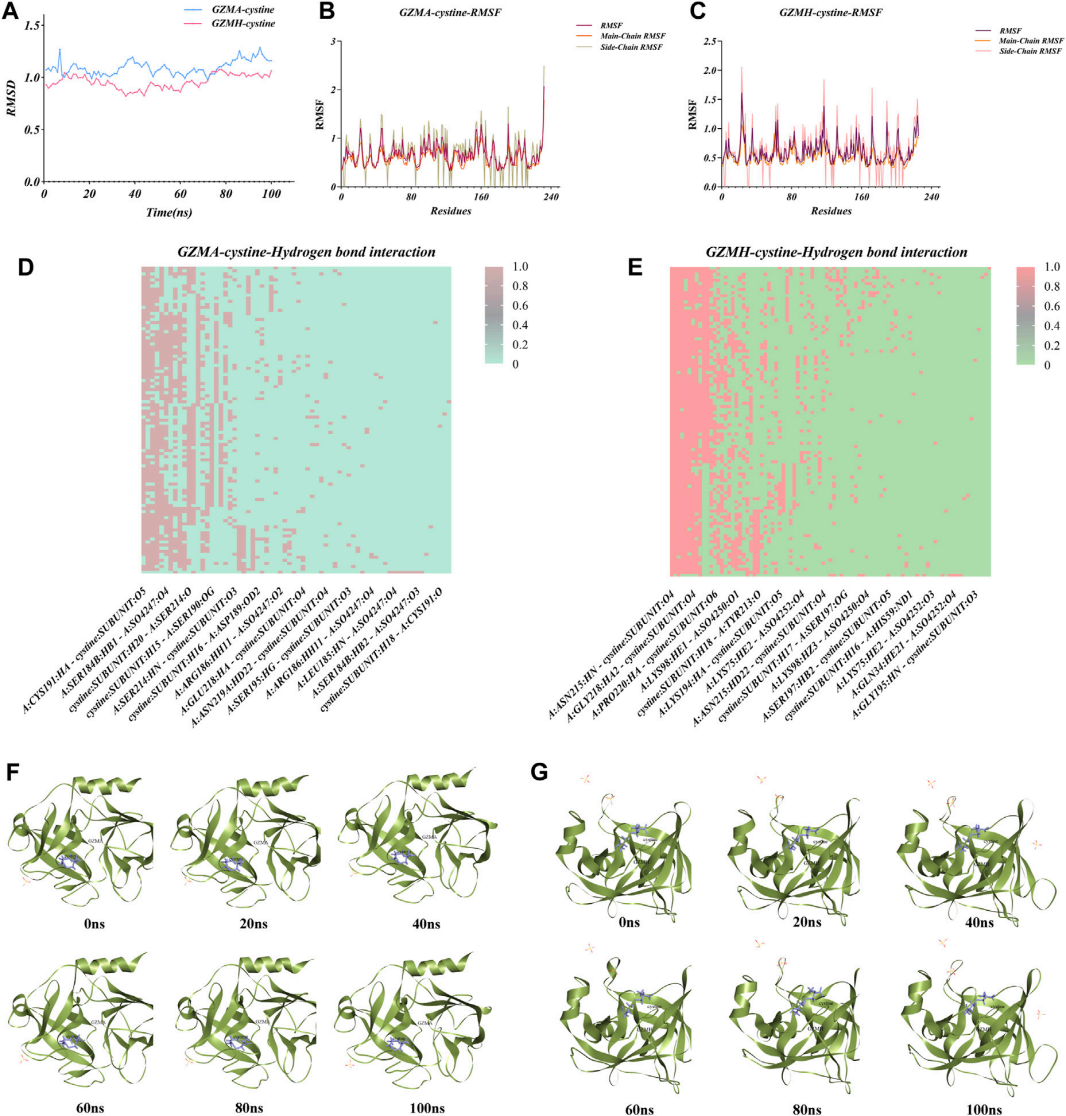
Analysis of atomic flow (MD) reenactments for protein-ligand complexes including cysteine with granzymes GZMA and GZMH. **(A)** Root-mean-square deviation (RMSD) directions throughout the MD reenactment for GZMA-cysteine and GZMH-cysteine complexes. The blue dashes speak to the RMSD values for the GZMA-cysteine complex, whereas pink shows the GZMH-cysteine complex, giving experiences into the soundness and conformational changes of each complex over time. **(B)** Plot of root-mean-square vacillation (RMSF) values for the GZMA-cysteine complex over the amino corrosive buildups, showing the adaptability and energetic developments of particular protein districts upon official with cysteine. **(C)** RMSF values for the GZMH-cysteine complex, additionally outlining the fluctuating parts of the protein structure and highlighting locales of intrigue that contribute to the authoritative soundness. **(D)** Warm outline of hydrogen bond interactions all through the MD reenactment for the GZMA-cysteine complex. The nearness and recurrence of hydrogen bonds between the protein and ligand are delineated over the reenactment time, giving a quantitative representation of the authoritative intuition. **(E)** Warm outline for the GZMH-cysteine complex, showing the hydrogen holding design and its solidness, with the escalation of the color relating to the quality and tirelessness of the hydrogen bonds. **(F)** Time-lapse depictions of the GZMA-cysteine complex, captured at 20 ns interims amid the MD reenactment, exhibiting the conformational states and the protein-ligand interaction at diverse time focuses. **(G)** Time-lapse snapshots for the GZMH-cysteine complex, were also taken at 20 ns intervals. These images allow for a visual comparison of the dynamic structural changes occurring during the MD simulation and can aid in the identification of stable interaction phases.

Molecular dynamics simulations were conducted on the two most stable complexes identified from the molecular docking studies, specifically the GZMA-cysteine and GZMH-cysteine complexes. These complexes exhibited conformational stability throughout the simulation duration, with the root-mean-square deviation (RMSD) reaching equilibrium at about 10–15 nanoseconds and fluctuation values maintaining below 0.2 nm. The calculated root-mean-square fluctuation (RMSF) values of the main and side chains for both complexes indicated that the proteins remained structurally stable post-binding to the metabolite cysteine. Notably, the RMSD values for the GZMA-cysteine complex primarily ranged from 0.976 to 1.29, with an average of 1.095, while the GZMH-cysteine complex exhibited fluctuations from 0.821 to 1.077, averaging at 0.959,547. These RMSD fluctuations had been within an affordable range, confirming the staleness of each complex at some stage in the molecular dynamics simulation process (Figures 6A–C). During the simulations, both the GZMA-cysteine and GZMH-cysteine complexes demonstrated robust binding within the active sites of their respective target proteins, facilitated by a variety of bond interactions, including hydrogen, hydrophobic, and Pi bonds (Figures 6D,E). The metabolic marker cysteine exhibited notable mobility within the GZMA protein structure, potentially leading to alterations in protein pore configurations and subsequent inhibition of protein function. Comparatively, the RMSF values and the variations in hydrogen bonding within the cysteine-GZMA complex were more pronounced than those within the cysteine-GZMH protein complex system (Figures 6F,G), suggesting that cysteine may exert a more substantial impact on GZMA. These findings shed light on the dynamic stability and potential inhibitory interactions of cysteine with these proteins, paving the way for future therapeutic strategies targeting these complexes in the treatment of diseases where GZMA and GZMH are implicated.

### 3.8 Cysteine reduces GZMA expression and enhances C2C12 cell activity

**FIGURE 7:**
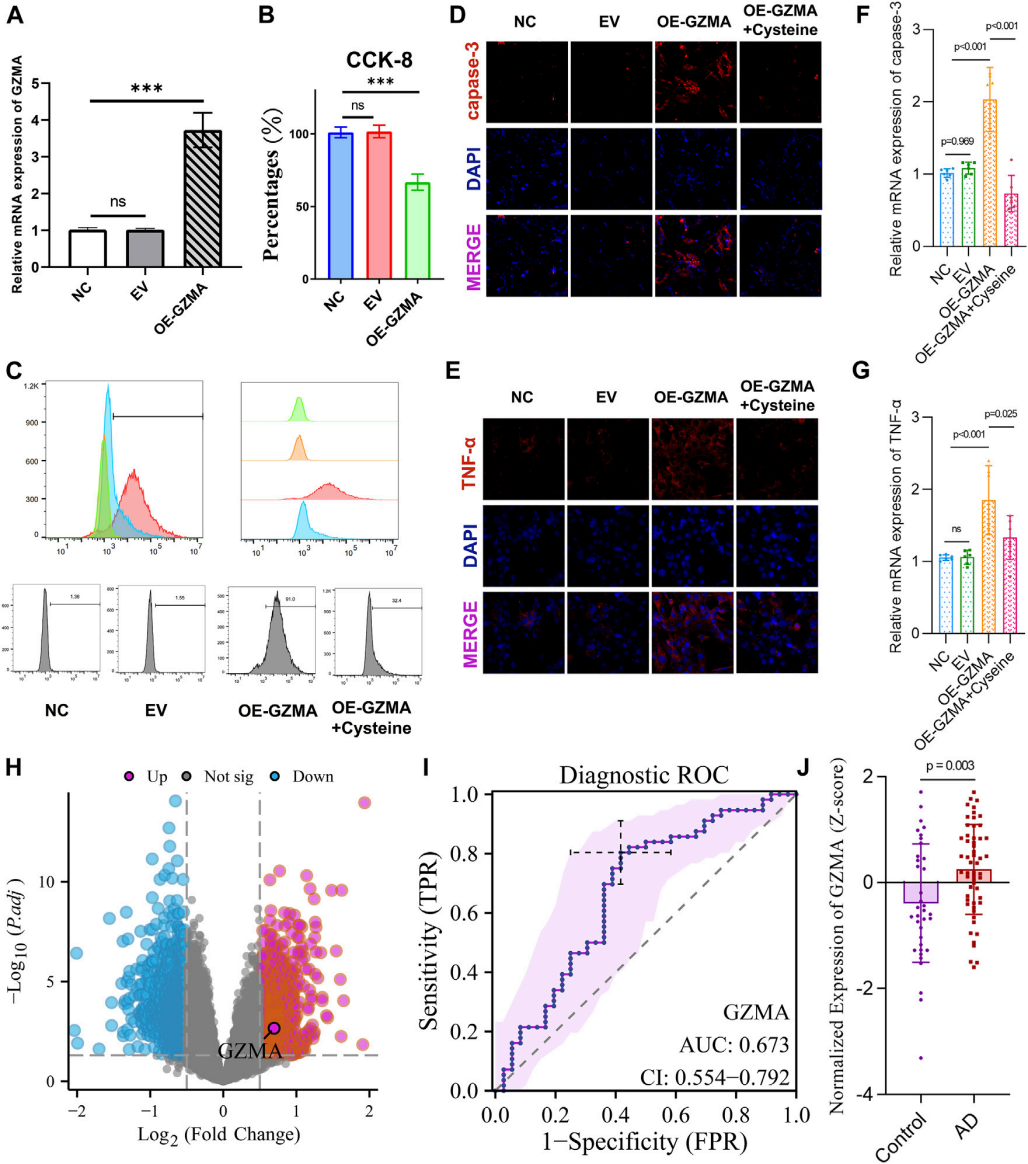
Cysteine reduces GZMA expression and enhances C2C12 cell activity. **(A)** Quantitative PCR results reveal a significant upregulation of GZMA mRNA expression in the OE-GZMA group compared to NC and EV, which is notably reduced upon cysteine treatment (*p < 0.001, ns = not significant). **(B)** CCK-8 assays show a significant reduction in cell viability in the OE-GZMA group as opposed to NC and EV, while cysteine intervention restores cell viability in OE-GZMA cells (*p < 0.001, ns = not significant). **(C)** Flow cytometry histograms depicting reactive oxygen species (ROS) levels indicate an increase in ROS upon GZMA overexpression, which is mitigated by cysteine treatment, as evidenced by the shift in fluorescence intensity. **(D)** Immunofluorescence images for caspase-3 in NC, EV, OE-GZMA, and OE-GZMA + cysteine-treated cells show an increase in caspase-3 immunofluorescence intensity in OE-GZMA, indicative of elevated apoptosis, which is reduced following cysteine intervention. DAPI staining identifies nuclei. **(E)** Immunofluorescence staining for TNF-α shows increased expression in OE-GZMA cells compared to NC and EV, while cysteine treatment lowers TNF-α levels, correlating with a reduction in inflammation. DAPI staining identifies nuclei. The trend in the immunofluorescence images suggests that GZMA overexpression leads to increased apoptosis and inflammation, which are alleviated by cysteine treatment. **(F)** Quantitative PCR results for caspase-3 demonstrate significant differences between groups, confirming elevated apoptosis in OE-GZMA cells, which is alleviated by cysteine treatment (*p < 0.001, ns = not significant). **(G)** Quantitative PCR results for TNF-α show significant differences between groups, indicating increased inflammation in OE-GZMA cells, which is reduced by cysteine treatment (*p < 0.001, ns = not significant). **(H)** Volcano plot reveals the differential expression of GZMA in AD patients compared to controls in the GSE122063 dataset, indicating significant upregulation. **(I)** ROC curve reveals the diagnostic capability of GZMA for AD, with an AUC of 0.673, CI: 0.554–0.792. **(J)** Z-score analysis shows significantly higher expression of GZMA in AD patients compared to controls (*p < 0.001).

Our study aimed to investigate the effect of cysteine on GZMA expression and C2C12 cell activity. Quantitative PCR analysis revealed a significant upregulation of GZMA mRNA expression in the OE-GZMA group compared to the NC and EV groups, which was notably reduced upon cysteine treatment (***p < 0.001, ns = not significant) (Figure 7A). This suggests that cysteine effectively downregulates GZMA overexpression. The CCK-8 assay results demonstrated a significant reduction in cell viability in the OE-GZMA group compared to NC and EV, whereas cysteine intervention restored cell viability in OE-GZMA cells (***p < 0.001, ns = not significant) (Figure 7B). This indicates that cysteine has a protective effect on C2C12 cells overexpressing GZMA. Flow cytometry histograms depicting ROS levels indicated an increase in ROS upon GZMA overexpression, which was mitigated by cysteine treatment, as evidenced by the shift in fluorescence intensity (Figure 7C). This demonstrates that cysteine can reduce oxidative stress in cells overexpressing GZMA. Immunofluorescence staining for caspase-3 and TNF-α was performed to assess the levels of apoptosis and inflammation. Caspase-3 immunofluorescence intensity increased in the OE-GZMA group, indicative of elevated apoptosis, but this increase was reduced following cysteine intervention (Figure 7D). Similarly, TNF-α expression was higher in OE-GZMA cells compared to NC and EV groups, while cysteine treatment lowered TNF-α levels, correlating with a reduction in inflammation (Figure 7E). The quantitative analysis confirmed these observations, with significant differences between groups, highlighting the alleviation of apoptosis and inflammation by cysteine treatment (***p < 0.001, ns = not significant) (Figures 7F,G). Overall, these results demonstrate that cysteine effectively reduces GZMA expression, decreases oxidative stress, and mitigates apoptosis and inflammation in C2C12 cells overexpressing GZMA. A volcano plot revealed the differential expression of GZMA in AD patients compared to controls in the GSE122063 dataset, indicating significant upregulation (Figure 7H). The ROC curve revealed the diagnostic capability of GZMA for AD, with an AUC of 0.673, CI: 0.554–0.792 (Figure 7I). Z-score analysis showed significantly higher expression of GZMA in AD patients compared to controls (Figure 7J). These findings suggest that GZMA could serve as a potential biomarker for AD diagnosis.

### 3.9 Comprehensive analysis of GZMA expression and its implications in cancer

**FIGURE 8:**
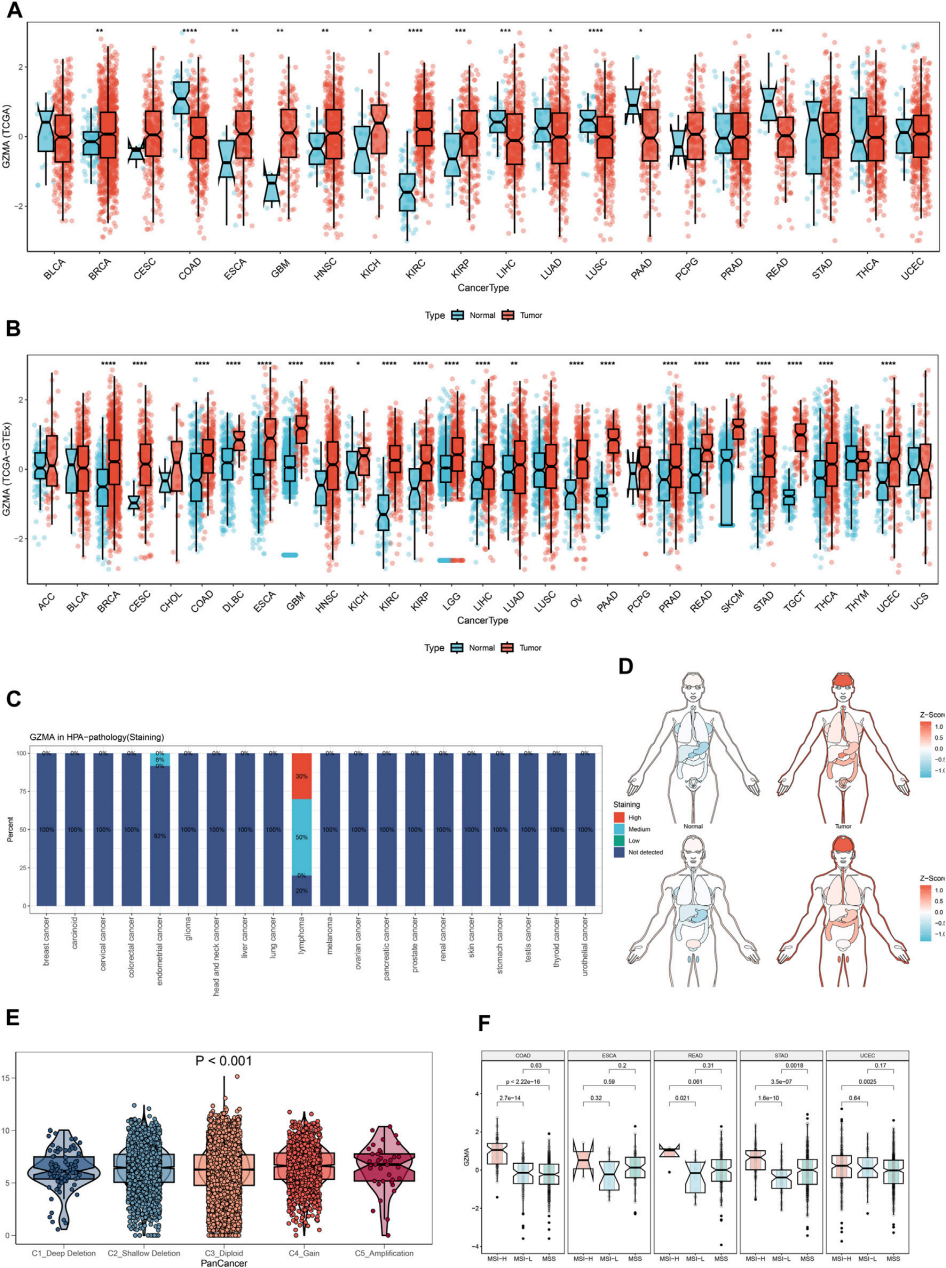
The Pan-Cancer Expression Landscape of GZMA. **(A, B)** Dysregulated expression of GZMA across multiple cancer types. The expression levels of GZMA are generally higher in tumor tissues compared to normal tissues. Each plot represents a different type of cancer with the x-axis indicating the cancer type and the y-axis showing the z-score normalized expression levels. Red box plots represent tumor groups, while blue box plots represent normal groups. Statistical significance is indicated where applicable (*p < 0.001). **(C)** The expression landscape of GZMA in various tissues measured in normalized transcripts per million (nTPM) from the Human Protein Atlas (HPA). The bar graph shows the differential expression of GZMA across a variety of tissues in both normal and tumor states. **(D)** Heatmap depicting the expression levels of GZMA in different parts of the human body. The color gradient indicates the level of expression, with red representing higher expression and blue representing lower expression. **(E)** Violin plot illustrating the differences in GZMA gene expression among various molecular subtypes. The x-axis represents different copy number alteration (CNA) categories such as deep deletion, shallow deletion, diploid normal, gain, and amplification, while the y-axis represents the expression levels. Statistical significance is indicated (*p* < 0.001). **(F)** Box plots showing the expression differences of GZMA across various molecular subtypes in different cancer types. Each plot corresponds to a specific cancer type, with the x-axis representing the molecular subtypes and the y-axis indicating the expression levels.

**FIGURE 9:**
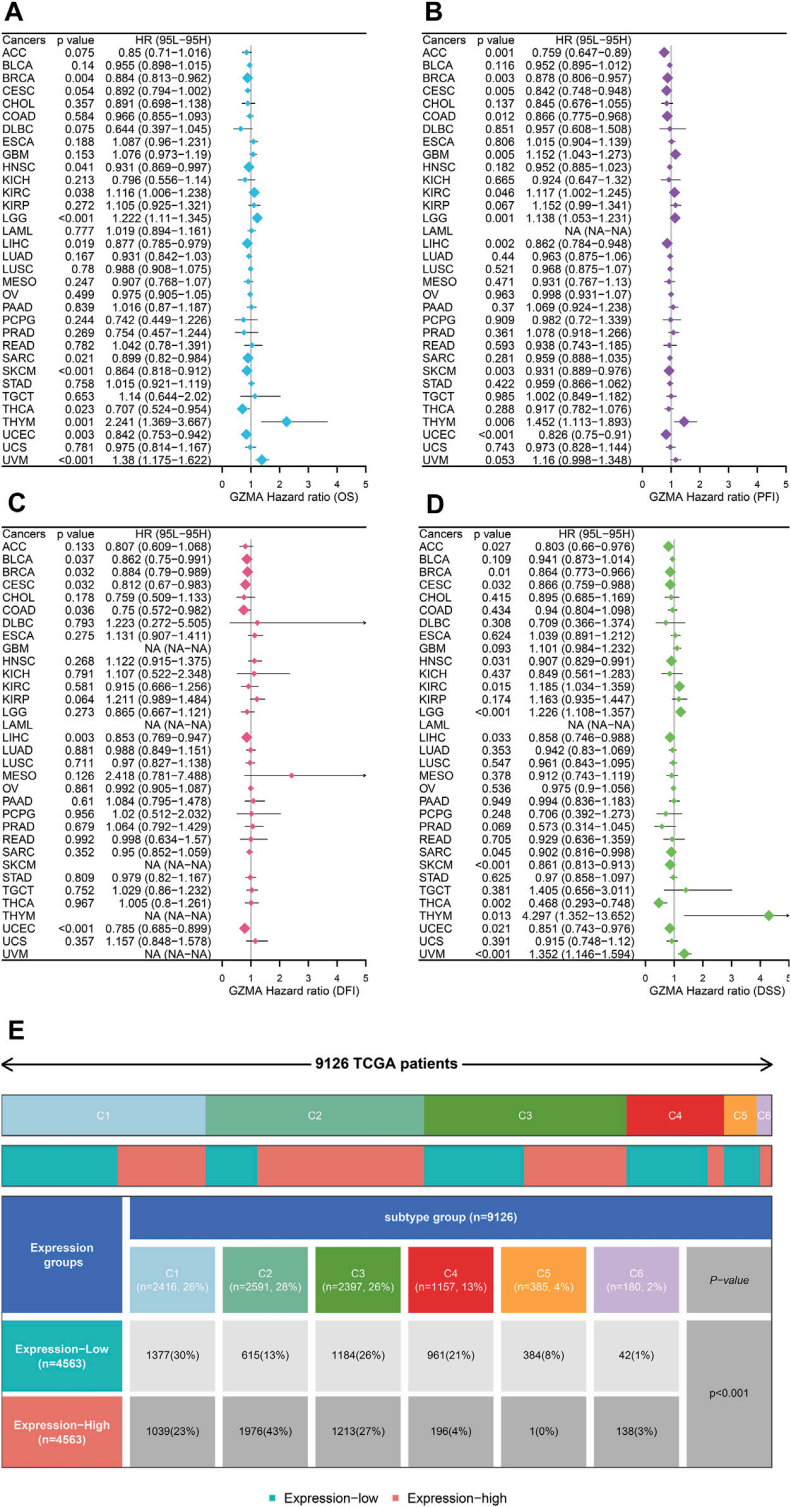
GZMA Pan-Cancer Survival and Immunity Analysis **(A–D)** Univariate survival analysis of GZMA expression across various cancers for four survival outcomes:**(A)** Overall Survival (OS): Hazard ratios (HR) with 95% confidence intervals (CI) are shown for each cancer type. Statistically significant results are marked, showing the impact of GZMA expression on OS. **(B)** Progression-Free Interval (PFI): Similar analysis as in **(A)** but for PFI, indicating the duration a patient remains free from disease progression. **(C)** Disease-Free Interval (DFI): Analysis focusing on the time after primary treatment that the patient remains free from any signs and symptoms of the cancer. **(D)** Disease-Specific Survival (DSS): Analysis showing the impact of GZMA expression on survival specific to the cancer being studied. For each panel **(A–D)**, data points represent the hazard ratios (HR) and 95% confidence intervals (CI) for the effect of GZMA expression on the respective survival outcomes across different cancer types. Statistically significant *p*-values (*p* < 0.05) are highlighted to indicate significant associations. **(E)** The distribution of GZMA expression levels across immune subtypes in 9126 TCGA patients. The upper part of the panel shows the proportion of each immune subtype (C1-C5) among the patient population. The lower part illustrates the distribution of patients with high and low GZMA expression within each immune subtype, along with associated *p*-values indicating the significance of the distribution differences.

In our study, we performed a complete evaluation of GZMA expression throughout more than one cancer type, revealing tremendous dysregulation in tumor tissues in comparison to everyday tissues. Figures 8A,B illustrate the better expression degrees of GMA in diverse cancers, with statistical importance (*p < 0.001). The Human Protein Chartbook (HPA) information in Figure 8C assists in authenticating these discoveries, indicating differential GZMA expression in different tissues. The heatmap in Figure 8D visualizes the expression levels over distinctive body parts, highlighting regions of raised GZMA expression. Figure 8E’s violin plot points out the expression contrasts among atomic subtypes categorized by duplicate number modifications (CNA), whereas Figure 8F’s box plots show these varieties over diverse cancer sorts. Survival analysis, portrayed in Figures 9A–D, looks at the effect of GZMA expression on general survival (OS), Progression-Free Interim (PFI), Disease-Free Interim (DFI), and Disease-Specific Survival (DSS), with noteworthy affiliations checked. At last, Figure 9E shows the conveyance of GZMA expression among safe subtypes in 9126 TCGA patients, giving insights into its role in cancer insusceptibility. These collectively emphasize GZMA’s potential as a pivotal biomarker and restorative target in cancer, advertising a point-by-point understanding of its expression scene and suggestions for persistent forecasting and treatment procedures.

### 3.10 GZMA single gene enrichment analysis results

Our comprehensive single gene enrichment analysis for GZMA uncovered critical intuition and pathways, including the development of protein-protein interaction (PPI) arrangement and GO/KEGG pathway enrichment. The GZMA interaction arrangement was created utilizing the STRING database (Supplementary Figure S3A) and incorporates different connection proteins categorized by their subcellular localization: cytosol, extracellular, layer, mitochondrion, core, and secretory pathway. These connections illustrate the known and predicted protein-protein interactions involving GZMA, highlighting its extensive involvement across different cellular compartments. The pathway enrichment analysis of GZMA, performed using GO and KEGG databases Supplementary Figure S3B), identified key pathways such as apoptosis, non-homologous end joining, and antigen processing and presentation, represented by the size and color of the dots—larger dots indicate higher gene ratios and darker colors represent more significant *p*-values. The GO/KEGG enrichment network diagram (Supplementary Figure S3C) illustrates the significant GO and KEGG terms associated with GZMA, with each line representing a connection to a relevant biological process, cellular component, or molecular function. This organized visualization illustrates the complex connections and pathways in which GZMA is included, emphasizing its multifaceted role in cellular forms. These investigations give nitty-gritty insights into the interaction and useful scene of GZMA, underscoring its significance in different organic pathways and its potential as a restorative target. The results are upheld by broad measurable analysis and graphical representation, guaranteeing clarity and comprehensiveness in understanding the importance of GZMA in cellular science.

### 3.11 GZMA single gene GSEA/GSVA enrichment analysis

**FIGURE 10:**
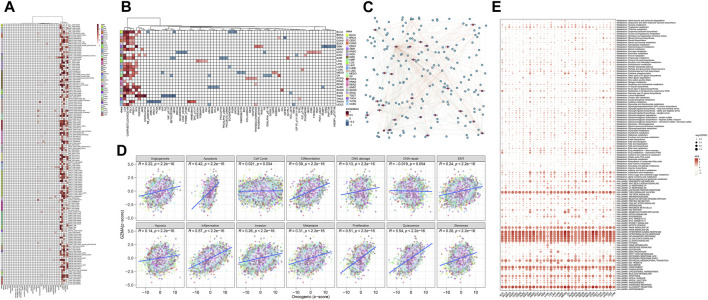
GZMA Single Gene GSEA/GSVA Enrichment Analysis. **(A)** GSEA Enrichment Analysis: This panel displays the Gene Set Enrichment Analysis (GSEA) results for GZMA, indicating the enrichment of specific gene sets associated with GZMA expression. The heatmap shows normalized enrichment scores (NES) with corresponding *p*-values for various gene sets. Significant enrichment is indicated through darker sun sunglasses of purple. **(B)** Expression of Immune Core Factors in Different Tumor Subtypes: This heatmap illustrates the differential expression of key immune elements throughout numerous tumor subtypes. Each row represents an immune factor, and every column represents a tumor subtype. The graduation scale represents the expression stages, with purple indicating better expression and blue indicating decreased expression. **(C)** Immune Core Factor Interaction Analysis: This community diagram suggests the interactions among immune middle elements. Nodes constitute man or woman immune elements, at the same time as edges imply interactions amongst them. The length of every node corresponds to the diploma of interplay, and part thickness represents interplay strength. **(D)** Enrichment Analysis of Multiple Gene Sets Comparing High and Low Expression Groups Using Cluster Profiler: Scatter plots display the consequences of enrichment evaluation completed through the cluster Profiler package. Each plot compares excessive and occasional expression corporations of GMA for numerous gene units, with axes representing enrichment ratings and statistical significance. **(E)** Immune Infiltration Expression Heatmap: This heatmap offers the expression stages of numerous immune molecular kinds throughout extraordinary samples. Rows constitute immune molecular kinds, at the same time as columns constitute man or woman samples. The graduation depth shows the extent of immune molecular infiltration, with purple representing better infiltration stages.

The analysis of GZMA single quality expression through Quality Set Enhancement Analysis (GSEA) and Quality Set Variety Analysis (GSVA) uncovered a few noteworthy discoveries. Figure 10A illustrates how the GSEA comes about for GZMA, showing the enhancement of particular quality sets related to GZMA expression, with noteworthy enhancement highlighted by darker shades of ruddy within the heatmap. Figure 10B illustrates the differential expression of key immune factors across various tumor subtypes, showing higher expression levels in red and lower levels in blue, underscoring variability in immune factor expression. Figure 10C provides a network diagram depicting the interactions among immune core factors, with nodes representing individual immune factors and edges indicating interaction strength, highlighting the complex interplay between these factors. Using the clusterProfiler package, enrichment analysis comparing high and low expression groups of GZMA for various gene sets is shown in Figure 10D through scatter plots, illustrating enrichment scores and statistical significance. Lastly, Figure 10E presents a heatmap of immune cell type expression levels across different samples, indicating levels of immune cell infiltration, with darker shades of red representing higher infiltration. These results collectively emphasize the critical role of GZMA in immune regulation and its association with various gene sets and immune factors, potentially informing therapeutic strategies and prognostic assessments in oncology.

## 4 Discussion

This study aims to investigate the impact of alternation in peripheral blood metabolites in patients with AD on POP and to examine the potential of multi-omics methodologies in clinical treatment. By combining traditional medicine and natural product therapies with contemporary scientific methods, we analyzed signaling pathways, gene expression profiles, and protein-metabolite interactions, with a specific emphasis on the roles of GZMA and cysteine in geriatric diseases, similar to studies on therapeutic targets for solid tumors and pharmacological mechanisms. In particular, natural compounds such as hesperidin, curcumin, and resveratrol have shown promise in modulating signaling pathways and gene expression, thus offering potential therapeutic benefits for AD and POP patients. The significance of cell death and metabolic regulation in disease progression is increasingly acknowledged, offering novel targets and strategies for pharmaceutical development (Zhang et al., 2024). The investigation of gene expression and regulatory mechanisms in diseases has been progressing, furnishing essential evidence for comprehending disease onset and progression (Li et al., 2024). This study utilized multi-omics analysis, encompassing metabolomics and transcriptomics, to evaluate variations in peripheral blood metabolites in AD patients and their influence on POP. We also conducted pan-cancer analysis and immune infiltration analysis of the core targets linking AD and POP, exploring their potential roles in tumor advancement and solid tumor pharmacology. The findings revealed that 47 distinct metabolites were linked to 9 crucial signaling pathways, including unsaturated fatty acid biosynthesis and amino acid metabolism. Extensive screening identified numerous DEGs, and subsequent GSEA indicated notable gene expression alternations in both AD and POP. Integrative network topology analysis of DEGs across multiple datasets identified central nodes in the AD-POP co-expression gene network. Functional analysis demonstrated that these genes are engaged in vital biological processes and pathways. Molecular docking studies revealed that cysteine strongly interacted with PTGS2 and GZMA proteins, while molecular dynamics simulations confirmed the stability of the resultant complexes. Further *in vitro* cell validation demonstrated that cysteine intervention effectively reduced ROS levels and safeguarded cell viability. GZMA is widely expressed in pan-cancer, linked with immune cells, and closely associated with the survival prognosis of cancer patients. The utilization of big data and bioinformatics in the identification and application of biomarkers is becoming increasingly important in disease diagnosis and prognosis evaluation (Liu et al., 2023).

In addition to the roles of GZMA and cysteine, this study highlights the potential therapeutic applications of natural compounds. Natural products have been explored for their therapeutic properties in various diseases, including neurodegenerative disorders and cancers. For example, compounds like hesperidin, curcumin, and resveratrol have shown promise in modulating signaling pathways and gene expression related to AD and POP (Villaflores et al., 2012; William Raja et al., 2023). These natural compounds may offer synergistic effects when combined with traditional and contemporary treatments, providing a holistic approach to disease management. Including information about related clinical trials and preclinical studies on these natural compounds would offer better context and background for the readers (Andrade et al., 2019; Bonsignore et al., 2021).

Dementia is the most common mental disorder in older adults, and AD is the most common type of dementia. As the population ages, dementia will significantly affect public health, healthcare delivery, and social security systems in countries around the world (Kalaria et al., 2008). POP is a disease of abnormal pelvic organ position and function due to weak pelvic floor supporting tissues, which can lead to stress urinary incontinence, urinary or defecation disorders, etc. It is more prevalent in older women and significantly impacts their quality of life, with 10%–20% of women potentially undergoing surgical treatment for POP during their lifetime (Giarenis and Robinson, 2014; Chung and Kim, 2018; Mattsson et al., 2020). It has been found that peripheral blood metabolite levels are altered to varying degrees in AD patients, and these changes in metabolite levels can worsen the progression of AD (Wang et al., 2020). Both AD and POP are prevalent in the elderly, and clinical observations have identified patients with a combination of both disorders. Research has indicated that neurological disorders can contribute to pelvic organ prolapse, and AD and POP share common biomarkers, such as Aβ42 and tau (Alzheimer’s Disease Neuroimaging Initiative et al., 2018). When POP occurred in AD patients, it may accelerate the progression of the disease due to cognitive dysfunction and significantly impact the prognosis of patients’ quality of life. Therefore, it is urgent to explore the molecular mechanism of the potential role between the two conditions, and it is of clinical guidance for AD patients with coexisting POP.

In this study, analysis of metabolomic data from peripheral blood of AD patients identified 47 differentially expressed marker metabolites, including arachidonic acid, docosahexaenoic acid, linoleic acid, adrenic acid, elaidic acid, palmitic acid, etc., mainly enriched in Biosynthesis of unsaturated fatty acids, alanine, aspartate and glutamate metabolism, linoleic acid metabolism, and nitrogen metabolism signaling pathways. Wang et al. found that metabolites such as linoleic acid and the metabolic pathway of biosynthesis of unsaturated fatty acids synthesis contribute to the diagnosis of AD and POP are expected to predict early cognitive impairment prior to the manifestation of clinical symptoms of AD (Wang et al., 2020). This is consistent with our research findings. Arachidonic acid is involved in the pathogenesis of Alzheimer’s disease. Arachidonic acid in food promotes the development of Alzheimer’s disease, and the imbalance of APOE ε 4 specificity of arachidonic acid is an important biomarker for preclinical AD (Thomas et al., 2016; Abdullah et al., 2017). Glutamate hyperactivation has been suggested to be involved in the pathophysiology of AD (Wang and Reddy, 2017; Lin et al., 2019). Metabolites and related signaling pathways, in addition to playing an important role in the development of AD, are also closely related to the pathogenesis of POP. Current studies on metabolism-related pathways in POP have focused on tissue metabolites, such as elastin metabolism in the vagina and fibroblast collagen metabolism in pelvic tissue (Zong et al., 2010; Li et al., 2017).

The biological processes involved mainly include pyrimidine-containing compound catabolic process, pyrimidine deoxyribonucleotide catabolic process, pyrimidine nucleotide catabolic process, etc. Abnormal pyrimidine metabolism is associated with the early pathology of AD (Zhao et al., 2021a). The pyrimidine biosynthetic pathway in the brain is protective in AD patients (Pesini et al., 2019). Desler et al. found a role for deoxyribonucleotides in the pathogenesis of AD, with dTTP levels in peripheral blood mononuclear cells (PBMC) being an indicator of relative cognitive changes (Desler et al., 2015). After enrichment analysis of KEGG pathways of differentially expressed genes in the peripheral blood of AD patients, a total of 2 pathways enriched by core targets were obtained, including Purine metabolism, Glycosaminoglycan biosynthesis-heparan sulfate/heparin. This is similar to previous findings, where Xiang et al. found that purine metabolism is involved in the pathogenesis of AD (Xiang et al., 2015). In the early stages of AD pathology, purine-related metabolites and their converting enzymes are altered in the frontal, parietal and temporal cortices, and stage- and region-dependent dysregulation of purine metabolism can occur during AD progression (Ansoleaga et al., 2015; Alonso-Andrés et al., 2018). Interaction of apoE with heparan sulfate proteoglycans (HSPGs) is associated with the pathogenesis of AD (Libeu et al., 2001). Previous studies have found that larger oligosaccharides, heparin and acetyl heparan sulfate polymers inhibit the toxicity of apoE peptides in AD (Bazin et al., 2002). One study found that P53 expression was downregulated and apoptosis proteins (Bax and Bad) expression was upregulated in POP patients (Bai et al., 2005; Zhao et al., 2017). Increased mitochondrial apoptosis may promote the pathological process of POP (Kim et al., 2013).

In this study, intersection analysis of diseases and Cytoscape topology revealed that PTGS2, GZMA, PLAUR, CD200, GZMH, and MMP19 were bridges between AD and POP, and the molecular functions of these genes are predominantly enriched in serine-type endopeptidase activity, serine-type peptidase activity, and serine hydrolase activity, which may be linked to the metabolism of substances *in vivo*. PTGS2 has been shown to play a significant role in the pathogenesis of AD (Michele et al., 2014). PTGS2, also known as cyclooxygenase 2 (COX-2), is a key enzyme in arachidonic acid metabolism and is upregulated in brain regions of AD patients, and the PTGS2 gene is a susceptibility gene for AD (Ma et al., 2008; Zhou et al., 2018). The dual action of dioxygenase and peroxidase possessed by PTGS2 may contribute to alterations in peripheral microvascular reactivity and tissue distribution (Feng et al., 2017; Zhou et al., 2018). Granzyme A (GZMA), the most abundant serine protease in the cytotoxic granules of killer cells, possesses pro-inflammatory activity and can activate a novel programmed cell death pathway (Hashimoto et al., 2009; Lieberman, 2010). GZMA may also be involved in muscle dysfunction through ALS-related signaling pathways. GZMA can be modulated by estrogen. POP patients have pelvic floor muscle dysfunction, and some studies suggest that estrogen is effective in the treatment of POP (Ismail et al., 2010; Karpuzoglu et al., 2014; Brandt and Janse van Vuuren, 2019; Zhao et al., 2021b). GZMH has been identified at high levels in CD8 + T cells of patients with HTLV-1 associated myelopathy/tropical spastic paralysis (HAM/TSP), which may migrate to the central nervous system (CNS) (Malta et al., 2013). GZMH is also involved in the regulation of immunity and is associated with cytotoxic functions (Bertucci et al., 2018; Fu et al., 2019). A bioinformatics analysis has shown that POP-associated DEGs enriched in immune response and apoptotic processes (Zhou et al., 2018). CD200, an anti-inflammatory glycoprotein expressed in neurons, T cells, and B cells, has been observed to enhance microglia-mediated Aβ clearance and neural differentiation in patients with AD, exhibiting potential therapeutic effects (Varnum et al., 2015). Previous studies have identified signaling pathways enriched for POP differential genes, including cytokine-cytokine interactions, suggesting a possible in the inflammatory response (Zhou et al., 2018). We hypothesize that CD200, being commonly associated with both AD and POP, could be a promising therapeutic target for patients with comorbid AD and POP. The PLAUR gene encodes the receptor for urokinase fibrinogen activator, known as PLAUR (uPAR) (Zhang et al., 2021). The pathological mechanisms involving uPAR-related pathways have been implicated in the development, function, and neurodegenerative pathologies such as AD in the central nervous system (Bruneau and Szepetowski, 2011). In addition, uPAR is involved in macrophage and neutrophil infiltration (Rijneveld et al., 2002). The degree of infiltration of activated mast cells and neutrophils was higher in POP tissues than in non-POP tissues (Zhao et al., 2020).

MMP19 has been found to be associated with neurodegenerative processes and related pathways, is one of the differential genes for early and late mild cognitive impairment. It is also associated with brain amyloid angiopathy, a common active process in AD progression (Tanskanen et al., 2011; Rempe et al., 2016; Brito et al., 2020). The interaction of MP-1 and MMP-3 gene polymorphisms of the MMMP family may contribute to the development of POP in some women, and MMP13 has also been found to promote the progression of POP (Zong et al., 2009; Skorupski et al., 2013). MMPs regulate the degradation of collagen (Liu et al., 2020; Yue et al., 2020). Functional failure of the vagina and its supporting tissues, one of the main means of supporting the pelvic organs, whose collagen content is related to tensile strength, will result in the descent of the pelvic organs into the vagina (Goh, 2003; Zong et al., 2009). MMP19, an important component of MMPs, may also play a role in promoting POP progression. Based on previous studies and the present findings, we suggest that PTGS2, GZMA, PLAUR, CD200, GZMH, and MMP19, as genes commonly associated with AD and POP, may lay the foundation for further exploration of specific mechanisms and therapeutic targets for the development of POP in AD patients.

Combined therapy has been found to have a significant effect on disease recovery (Cho et al., 2023; Zhang et al., 2023). In recent years, studies have shown that through the regulation of specific biomolecules and the application of specific compounds, such as hesperidin and curcumin, there is potential to address various biological responses (Du and Liu, 2024). Clinical trials involving natural compounds like resveratrol and curcumin have shown encouraging results in improving cognitive function and reducing oxidative stress in AD patients, providing a strong basis for their potential application in POP treatment. Moreover, the use of big data analysis and bioinformatics has demonstrated prospects in improving prognosis and health management (Yao et al., 2024). Molecular docking methods and molecular dynamics (MD) simulations can determine the binding energy between the ligand and the receptor (Kordzadeh et al., 2020). Molecular docking allows the analysis of a large number of ligands, but the ignorance of the dynamics of ligands and receptors reduces the accuracy of this approach (Ravindranathan et al., 2010). Molecular dynamics simulations effectively validate the docking results. The combination of the two can provide valuable insights (Jiang et al., 2018). In this study, we found that the structures of GZMA-cysteine complex and GZMH-cysteine complex were in equilibrium after simulation and in a stable state throughout the MD simulation, indicating that the peripheral blood metabolite cysteine binds most strongly to the core protein GZMA/GZMH in AD patients. The extracellular redox environment is mainly determined by redox-coupled cysteine/cysteine. As age increases, the extracellular redox environment shifts towards oxidation after middle age. Reducing redox potential by controlling the extracellular redox environment has neuroprotective effects on both aging and AD-like neurons (Ghosh and Brewer, 2014). Cysteine metabolism is involved in AD progression. The redox of extracellular cysteine/cysteine may affect AD progression by altering free NADH levels and redox status (Dong et al., 2019). Previous studies have identified enhanced expression of cysteine/glutamate transport proteins as a feature of AD (Ashraf et al., 2020). The diagnostic model constructed by Cysteine and CPB2 also performs well in the diagnosis of AD (Yang et al., 2022). Redox-related pathways are also involved in the development of POP. In POP patients, the oxidative and antioxidant balance in the pelvic support structures is dysregulated and the expression of mitochondrial respiratory chain complexes in the vaginal wall is reduced (Li et al., 2013; Alujević et al., 2018). In addition, immune response-related pathways also play a role in POP progression (Zhou et al., 2018). The degree of infiltration of activated mast cells and neutrophils was higher in POP tissues than in non-POP tissues (Zhao et al., 2020). Cysteine is involved in the immune response of dendritic cells, and neutrophils express xCT, a transporter protein that mediates the entry of extracellular cysteine into cells (Tanaka et al., 2015). Cysteine is essential in suppressing the inflammatory response. The inflammatory response is the main mechanism in the pathogenesis of POP (Li Y. et al., 2021). Therefore, the study of altered cysteine levels is important to elucidate the specific mechanism of the effect of AD peripheral metabolic disorders on the developmental process of POP.

Clinical trials have investigated the use of cysteine and its derivatives in various medical conditions. In preterm infants, high-dose cysteine supplementation did not increase glutathione synthesis (Te Braake et al., 2009). For acute respiratory distress syndrome, N-acetylcysteine (NAC) and procysteine showed potential benefits in reducing the duration of acute lung injury (Bernard et al., 1997). Cysteine treatment improved light tolerance in erythropoietic protoporphyria patients. NAC demonstrated promise in reducing prematurity-related morbidities in newborns exposed to intrauterine infection (Buhimschi et al., 2021). However, NAC showed no significant benefit in preserving lung function in idiopathic pulmonary fibrosis patients (The Idiopathic Pulmonary Fibrosis Clinical Research Network et al., 2014). In schizophrenia, NAC as an adjunct therapy improved symptoms and functioning (Berk et al., 2008). NAC also showed potential as a safe adjuvant treatment in acute organophosphorus pesticide poisoning, reducing atropine requirements (El Ebiary et al., 2016). These studies highlight the diverse applications of cysteine and its derivatives in clinical settings.

Given the wide range of therapeutic effects observed with cysteine and its derivatives, these compounds offer promising avenues for addressing the metabolic and inflammatory dysregulation observed in both AD and POP. For instance, cysteine’s potential in reducing oxidative stress and modulating immune responses can be leveraged in managing AD, while its role in collagen metabolism and tissue repair may offer therapeutic benefits for POP (Hara et al., 2017; Paul et al., 2018). Moreover, the ability of cysteine and related compounds to influence apoptotic pathways and immune responses suggests potential applications in cancer treatment, especially in mitigating tumor progression and enhancing patient prognosis. Therefore, the integration of cysteine-based therapies into the treatment regimens for AD, POP, and cancer could provide comprehensive strategies for managing these complex conditions.

Granzymes (Gzm) are a group of serine proteases stored in cytotoxic lymphocyte granules and be involved in apoptosis and other non-apoptotic immune-related effects such as ECM remodeling, cytokine regulation, and killing of pathogens through phagosome production (Ikram et al., 2021). The pathogenesis of AD involves hippocampal apoptosis (Hu et al., 2017). Apoptosis of the uterine ligament cells and mitochondrial apoptosis were also present in the POP pathological process (Kim et al., 2013; Zhao et al., 2017). In addition, it has been found that POP differential gene enrichment signaling pathways include cytokine-cytokine interactions and molecular changes related to extracellular matrix (ECM) organization (Zhou et al., 2018; Li Y. et al., 2021). In our investigation, we postulated that dysregulated cysteine levels in AD patients could influence the progression of POP by modulating the expression of GZMA/GZMH proteins. This metabolic alteration may affect apoptosis and other related pathways, proposing cysteine and the GZMA/GZMH protein targets as potential novel therapeutic avenues for managing AD-POP comorbidity. Natural compounds like hesperidin and resveratrol, which have shown efficacy in modulating these pathways in other studies, could be explored for their potential therapeutic effects in AD-POP comorbidity (Auti and Kulkarni, 2017; Piccialli et al., 2022). This study has the potential to predict the onset of POP in AD patients before significant clinical symptoms appear using metabolomics methods. This marks a significant progress in the development of key targets for predicting the occurrence and development of POP in AD patients.

The significance of transcriptomics research lies in its ability to provide new insights into cellular heterogeneity within intricate biological processes (Smith et al., 2023; Wu et al., 2023). The study of gene expression and regulatory mechanisms in diseases has been continually expanding, offering crucial evidence for understanding the occurrence and development of diseases (Mei et al., 2024; Sonnaila and Agrawal, 2024). Our study delved into the expression and immune infiltration characteristics of the GZMA gene in pan cancer cells, revealing its abnormal expression in tumors and closely related to various immune cells. In recent years, researchers have found a link between GZMA and tumorigenesis. GZMA participates in immune monitoring of tumor cells by its expression in cytotoxic T lymphocytes and natural killer cells. These cells can identify and eliminate malignant entities, with GZMA assuming a pivotal role. Moreover, GZMA may influence the survival and death of tumor cells within the tumor microenvironment. For example, GZMA can penetrate tumor cells through perforin-mediated pathways, triggering cell death programs. Some studies suggest that GZMA could be a potential target for cancer therapy. Manipulating GZMA activity or its expression levels within the tumor microenvironment could pave the way for innovative cancer therapeutics (Huo et al., 2023). Furthermore, GZMA can induce pyroptosis, an immune-related cell death process, by cleaving Gasdermin B (GSDMB) protein in tumor cells, which may help limit tumor progression (Huo et al., 2023).

Modern bioinformatics and big data technologies are increasingly important in disease diagnosis, prognosis assessment, and treatment (Huang L. et al., 2022; Guo et al., 2023). Additionally, by improving drug delivery systems and utilizing nanotechnology, the targeting and therapeutic effects of drugs can be significantly enhanced (Nittayacharn et al., 2024). Our findings also resonate with the long-standing tradition of using natural remedies and products in healthcare. Combining traditional natural product therapies with modern multi omics research changes our understanding of these practices. This comprehensive approach can provide valuable insights into how these traditional therapies affect gene expression and genetic susceptibility in disease prevention and management.

While the current study has laid the groundwork at the bioinformatics level, utilizing databases for the exploration, experimental validations, and clinical studies are planned for the future. Furthermore, the application of newly synthesized nanocomposites in cancer treatment has made significant progress, suggesting that future research could consider further integration of material and drug research (Safarkhani et al., 2024; Wang et al., 2024). These steps are crucial for contributing to the early detection and intervention in the progression of AD, especially where POP is a potential complication.

## 5 Conclusion

In conclusion, our comprehensive transcriptomic and metabolomic analyses, leveraging data from the GEO database, have revealed significant alterations in the peripheral blood biomarker cysteine among AD patients. These alterations appear to modulate the expression of transmembrane receptor proteins GZMA/GZMH, offering a potentially promising therapeutic target for POP in the context of AD. These findings not only deepen our comprehension of the metabolic interplay between AD and POP but also highlight the utility of metabolomics in the early identification and therapeutic targeting of POP within the AD patient cohort.

## Data Availability

The data presented in the study are deposited in the GEO repository, accession number: GSE97760 and GSE168813.

## Abbreviations

AD, Alzheimer’s disease; POP, pelvic organ prolapse; GO, Gene ontology; KEGG, Kyoto Encyclopedia of Genes and Genomes; PTGS2, Prostaglandin Endoperoxide Synthase 2; MMP19, Matrix metallopeptidase 19; GZMA, Granzyme A; GZMH, Granzyme H.

## References

[B1] Abdullah L. Evans J. E. Emmerich T. Crynen G. Shackleton B. Keegan A. P. (2017). APOE ε4 specific imbalance of arachidonic acid and docosahexaenoic acid in serum phospholipids identifies individuals with preclinical Mild Cognitive Impairment/Alzheimer’s Disease. Aging (Albany NY) 9, 964–985. 10.18632/aging.101203 28333036 PMC5391242

[B2] Alonso-Andrés P. Albasanz J. L. Ferrer I. Martín M. (2018). Purine-related metabolites and their converting enzymes are altered in frontal, parietal and temporal cortex at early stages of Alzheimer’s disease pathology: purine-related Metabolites and their Converting Enzymes in AD. Brain Pathol. 28, 933–946. 10.1111/bpa.12592 29363833 PMC8028663

[B3] Alujević J. I. Jakus D. Marinović J. Ćavar M. Banić I. Vilović K. (2018). Expression of mitochondrial respiratory chain complexes in the vaginal wall in postmenopausal women with pelvic organ prolapse. Gynecol. Obstet. Invest 83, 487–492. 10.1159/000480236 28850957

[B4] Alzheimer’s Disease Neuroimaging Initiative Maxwell T. J. Corcoran C. del-Aguila J. L. Budde J. P. Deming Y. Cruchaga C. (2018). Genome-wide association study for variants that modulate relationships between cerebrospinal fluid amyloid-beta 42, tau, and p-tau levels. Alz Res. Ther. 10, 86. 10.1186/s13195-018-0410-y PMC6114488 30153862

[B5] Andrade S. Nunes D. Dabur M. Ramalho M. J. Pereira M. C. Loureiro J. A. (2023). Therapeutic potential of natural compounds in neurodegenerative diseases: insights from clinical trials. Pharmaceutics 15, 212. 10.3390/pharmaceutics15010212 36678841 PMC9860553

[B6] Andrade S. Ramalho M. J. Loureiro J. A. Pereira M. D. C. (2019). Natural compounds for alzheimer’s disease therapy: a systematic review of preclinical and clinical studies. Int. J. Mol. Sci. 20, 2313. 10.3390/ijms20092313 31083327 PMC6539304

[B7] Ansoleaga B. Jové M. Schlüter A. Garcia-Esparcia P. Moreno J. Pujol A. (2015). Deregulation of purine metabolism in Alzheimer’s disease. Neurobiol. Aging 36, 68–80. 10.1016/j.neurobiolaging.2014.08.004 25311278

[B8] Ashraf A. Jeandriens J. Parkes H. G. So P.-W. (2020). Iron dyshomeostasis, lipid peroxidation and perturbed expression of cystine/glutamate antiporter in Alzheimer’s disease: evidence of ferroptosis. Redox Biol. 32, 101494. 10.1016/j.redox.2020.101494 32199332 PMC7083890

[B9] Auti S. T. Kulkarni Y. A. (2017). A systematic review on the role of natural products in modulating the pathways in Alzheimer’s disease. Int. J. Vitam. Nutr. Res. 87, 99–116. 10.1024/0300-9831/a000405 30010515

[B10] Bai S. W. Chung D. J. Yoon J. M. Shin J. S. Kim S. K. Park K. H. (2005). Roles of estrogen receptor, progesterone receptor, p53 and p21 in pathogenesis of pelvic organ prolapse. Int. Urogynecol J. 16, 492–496. 10.1007/s00192-005-1310-9 15915319

[B11] Bazin H. G. Marques M. A. Owens A. P. Linhardt R. J. Crutcher K. A. (2002). Inhibition of apolipoprotein E-related neurotoxicity by glycosaminoglycans and their oligosaccharides. Biochemistry 41, 8203–8211. 10.1021/bi025817e 12069613

[B12] Berk M. Copolov D. Dean O. Lu K. Jeavons S. Schapkaitz I. (2008). N-acetyl cysteine as a glutathione precursor for schizophrenia—a double-blind, randomized, placebo-controlled trial. Biol. Psychiatry 64, 361–368. 10.1016/j.biopsych.2008.03.004 18436195

[B13] Bernard G. R. Wheeler A. P. Arons M. M. Morris P. E. Paz H. L. Russell J. A. (1997). A trial of antioxidants N-acetylcysteine and procysteine in ARDS. The Antioxidant in ARDS Study Group. Chest 112, 164–172. 10.1378/chest.112.1.164 9228372

[B14] Bertucci F. Finetti P. Simeone I. Hendrickx W. Wang E. Marincola F. M. (2018). The immunologic constant of rejection classification refines the prognostic value of conventional prognostic signatures in breast cancer. Br. J. Cancer 119, 1383–1391. 10.1038/s41416-018-0309-1 30353048 PMC6265245

[B15] Blasko I. Defrancesco M. Oberacher H. Loacker L. Kemmler G. Marksteiner J. (2021). Plasma phosphatidylcholines and vitamin B12/folate levels are possible prognostic biomarkers for progression of Alzheimer’s disease. Exp. Gerontol. 147, 111264. 10.1016/j.exger.2021.111264 33516907

[B16] Bonsignore G. Patrone M. Grosso F. Martinotti S. Ranzato E. (2021). Cancer therapy challenge: it is time to look in the “St. Patrick’s well” of the nature. IJMS 22, 10380. 10.3390/ijms221910380 34638721 PMC8508794

[B17] Brækken I. H. Majida M. Engh M. E. Bø K. (2010). Can pelvic floor muscle training reverse pelvic organ prolapse and reduce prolapse symptoms? An assessor-blinded, randomized, controlled trial. Am. J. Obstetrics Gynecol. 203, 170.e1–170.e7. 10.1016/j.ajog.2010.02.037 20435294

[B18] Brandt C. Janse van Vuuren E. C. (2019). Dysfunction, activity limitations, participation restriction and contextual factors in South African women with pelvic organ prolapse. South Afr. J. Physiother. 75, 933. 10.4102/sajp.v75i1.933 PMC6407468 30863799

[B19] Brito L. M. Ribeiro-Dos-Santos Â. Vidal A. F. de Araújo G. S. (2020). Differential expression and miRNA-gene interactions in early and late mild cognitive impairment. Biol. (Basel) 9, E251. 10.3390/biology9090251 PMC7565463 32872134

[B20] Bruneau N. Szepetowski P. (2011). The role of the urokinase receptor in epilepsy, in disorders of language, cognition, communication and Behavior, and in the central nervous system. CPD 17, 1914–1923. 10.2174/138161211796718198 21711233

[B21] Buhimschi C. S. Bahtiyar M. O. Zhao G. Abdelghany O. Schneider L. Razeq S. A. (2021). Antenatal N-acetylcysteine to improve outcomes of premature infants with intra-amniotic infection and inflammation (Triple I): randomized clinical trial. Pediatr. Res. 89, 175–184. 10.1038/s41390-020-01106-w 32818949 PMC7451831

[B22] Bui T. T. Nguyen T. H. (2017). Natural product for the treatment of Alzheimer’s disease. J. Basic Clin. Physiology Pharmacol. 28, 413–423. 10.1515/jbcpp-2016-0147 28708573

[B23] Butler M. S. (2008). Natural products to drugs: natural product-derived compounds in clinical trials. Nat. Prod. Rep. 25, 475–516. 10.1039/b514294f 18497896

[B24] Chen B. Guo J. Ye H. Wang X. Feng Y. (2024a). Role and molecular mechanisms of SGLT2 inhibitors in pathological cardiac remodeling (Review). Mol. Med. Rep. 29, 73. 10.3892/mmr.2024.13197 38488029 PMC10955520

[B25] ChenY.ChenX.LuoZ.KangX.GeY.WanR. (2024b). Exercise-Induced reduction of IGF1R sumoylation attenuates neuroinflammation in APP/PS1 transgenic mice. J. Adv. Res., S2090123224001279. 10.1016/j.jare.2024.03.025 38565402

[B26] Chen Y. Kang X. Tao J. Zhang Y. Ying C. Lin W. (2019). Reliability of synovial fluid alpha-defensin and leukocyte esterase in diagnosing periprosthetic joint infection (PJI): a systematic review and meta-analysis. J. Orthop. Surg. Res. 14, 453. 10.1186/s13018-019-1395-3 31856885 PMC6921602

[B27] Chen Y. Luo Z. Lin J. Qi B. Sun Y. Li F. (2022b). Exploring the potential mechanisms of melilotus officinalis (L.) pall. In chronic muscle repair patterns using single cell receptor-ligand marker analysis and molecular dynamics simulations. Dis. Markers 2022, 9082576. 10.1155/2022/9082576 35692879 PMC9177293

[B28] Chen Y. Sun Y. Luo Z. Chen X. Wang Y. Qi B. (2022a). Exercise modifies the transcriptional regulatory features of monocytes in alzheimer’s patients: a multi-omics integration analysis based on single cell technology. Front. Aging Neurosci. 14, 881488. 10.3389/fnagi.2022.881488 35592698 PMC9110789

[B29] Chin C.-H. Chen S.-H. Wu H.-H. Ho C.-W. Ko M.-T. Lin C.-Y. (2014). cytoHubba: identifying hub objects and sub-networks from complex interactome. BMC Syst. Biol. 8, S11. 10.1186/1752-0509-8-S4-S11 25521941 PMC4290687

[B30] Cho J. Ryu S. Lee S. Kim J. Park J.-Y. Kwon H.-S. (2023). Clozapine-Induced chemogenetic neuromodulation rescues post-stroke deficits after chronic capsular infarct. Transl. Stroke Res. 14, 499–512. 10.1007/s12975-022-01059-8 35809218 PMC10300150

[B31] Chung S.-H. Kim W. B. (2018). Various approaches and treatments for pelvic organ prolapse in women. J. Menopausal Med. 24, 155–162. 10.6118/jmm.2018.24.3.155 30671407 PMC6336571

[B32] Desler C. Frederiksen J. H. Angleys M. Maynard S. Keijzers G. Fagerlund B. (2015). Increased deoxythymidine triphosphate levels is a feature of relative cognitive decline. Mitochondrion 25, 34–37. 10.1016/j.mito.2015.09.002 26408413 PMC5176333

[B33] Dong Y. Sameni S. Digman M. A. Brewer G. J. (2019). Reversibility of age-related oxidized free NADH redox states in alzheimer’s disease neurons by imposed external cys/CySS redox shifts. Sci. Rep. 9, 11274. 10.1038/s41598-019-47582-x 31375701 PMC6677822

[B34] Du Y. Liu H. (2024). Exercise-induced modulation of miR-149-5p and MMP9 in LPS-triggered diabetic myoblast ER stress: licorice glycoside E as a potential therapeutic target. Tradit. Med. Res. 9, 45. 10.53388/TMR20230121002

[B35] El Ebiary A. A. Elsharkawy R. E. Soliman N. A. Soliman M. A. Hashem A. A. (2016). *N* ‐acetylcysteine in acute organophosphorus pesticide poisoning: a randomized, clinical trial. Basic Clin. Pharma Tox 119, 222–227. 10.1111/bcpt.12554 26786042

[B36] Feng J. Anderson K. Liu Y. Singh A. K. Ehsan A. Sellke F. W. (2017). Cyclooxygenase 2 contributes to bradykinin-induced microvascular responses in peripheral arterioles after cardiopulmonary bypass. J. Surg. Res. 218, 246–252. 10.1016/j.jss.2017.05.086 28985857 PMC5649638

[B37] Fu Y.-J. Yan Y.-Q. Zheng X. Shi S.-S. Wu S. Jiang Z.-Y. (2019). Effects of Xinjiaxiangruyin on the TLR7 pathway in influenza virus-infected lungs of mice housed in a hygrothermal environment. Chin. Med. 14, 39. 10.1186/s13020-019-0256-7 31572491 PMC6764144

[B38] Gao S. Shi X. Yue C. Chen Y. Zuo L. Wang S. (2024). Comprehensive analysis of competing endogenous RNA networks involved in the regulation of glycolysis in clear cell renal cell carcinoma. Oncologie 0, 0. 10.1515/oncologie-2024-0074

[B39] Ghosh D. Brewer G. J. (2014). External cys/cySS redox state modification controls the intracellular redox state and neurodegeneration via akt in aging and alzheimer’s disease mouse model neurons. JAD 42, 313–324. 10.3233/JAD-132756 24844688 PMC6099062

[B40] Giarenis I. Robinson D. (2014). Prevention and management of pelvic organ prolapse. Prime Rep. 6, 77. 10.12703/P6-77 PMC4166938 25343034

[B41] Goh J. T. (2003). Biomechanical and biochemical assessments for pelvic organ prolapse. Curr. Opin. Obstetrics Gynecol. 15, 391–394. 10.1097/00001703-200310000-00007 14501242

[B42] Gulaj E. Pawlak K. Bien B. Pawlak D. (2010). Kynurenine and its metabolites in Alzheimer’s disease patients. Adv. Med. Sci. 55, 204–211. 10.2478/v10039-010-0023-6 20639188

[B43] Guo Z. Yu X. Zhao S. Zhong X. Huang D. Feng R. (2023). SIRT6 deficiency in endothelial cells exacerbates oxidative stress by enhancing HIF1α accumulation and H3K9 acetylation at the Ero1α promoter. Clin. & Transl. Med 13, e1377. 10.1002/ctm2.1377 37598403 PMC10440057

[B44] Hagen S. Stark D. Glazener C. Sinclair L. Ramsay I. (2009). A randomized controlled trial of pelvic floor muscle training for stages I and II pelvic organ prolapse. Int. Urogynecol J. 20, 45–51. 10.1007/s00192-008-0726-4 18806910

[B45] Hara Y. Fillit H. M. Dacks P. A. McKeehan N. (2017). Evaluation of the neuroprotective potential of n-acetylcysteine for prevention and treatment of cognitive aging and dementia. J. Prev. Alz Dis. 4, 201–206. 10.14283/jpad.2017.22 29182711

[B46] Hashimoto Y. Kurita M. Aiso S. Nishimoto I. Matsuoka M. (2009). Humanin inhibits neuronal cell death by interacting with a cytokine receptor complex or complexes involving CNTF receptor alpha/WSX-1/gp130. MBoC 20, 2864–2873. 10.1091/mbc.e09-02-0168 19386761 PMC2695794

[B47] Hu X. Song C. Fang M. Li C. (2017). Simvastatin inhibits the apoptosis of hippocampal cells in a mouse model of Alzheimer’s disease. Exp. Ther. Med. 15, 1795–1802. 10.3892/etm.2017.5620 29434767 PMC5776644

[B48] Huang J. Lin W. Sun Y. Wang Q. He S. Han Z. (2022a). Quercetin targets VCAM1 to prevent diabetic cerebrovascular endothelial cell injury. Front. Aging Neurosci. 14, 944195. 10.3389/fnagi.2022.944195 36118693 PMC9475220

[B49] Huang L. Liu P. Yang Q. Wang Y. (2022b). The KRAB domain‐containing protein ZFP961 represses adipose thermogenesis and energy expenditure through interaction with PPAR *α* . Adv. Sci. 9, 2102949. 10.1002/advs.202102949 PMC8805557 34747141

[B50] Huo Q. Ning L. Xie N. (2023). Identification of GZMA as a potential therapeutic target involved in immune infiltration in breast cancer by integrated bioinformatical analysis. BCTT 15, 213–226. 10.2147/BCTT.S400808 PMC10013577 36926265

[B51] Huo Z. Yu L. Yang J. Zhu Y. Bennett D. A. Zhao J. (2020). Brain and blood metabolome for Alzheimer’s dementia: findings from a targeted metabolomics analysis. Neurobiol. Aging 86, 123–133. 10.1016/j.neurobiolaging.2019.10.014 31785839 PMC6995427

[B52] Ikram S. Ahmad F. Ahmad J. Durdagi S. (2021). Screening of small molecule libraries using combined text mining, ligand- and target-driven based approaches for identification of novel granzyme H inhibitors. J. Mol. Graph. Model. 105, 107876. 10.1016/j.jmgm.2021.107876 33744783

[B53] Iqubal A. Rahman S. O. Ahmed M. Bansal P. Haider M. R. Iqubal M. K. (2021). Current quest in natural bioactive compounds for alzheimer’s disease: multi-targeted-designed-ligand based approach with preclinical and clinical based evidence. CDT 22, 685–720. 10.2174/1389450121999201209201004 33302832

[B54] Ismail S. I. Bain C. Hagen S. (2010). Oestrogens for treatment or prevention of pelvic organ prolapse in postmenopausal women. Cochrane Database Syst. Rev., CD007063. 10.1002/14651858.CD007063.pub2 20824855 PMC13590214

[B55] Jelovsek J. E. Barber M. D. Brubaker L. Norton P. Gantz M. Richter H. E. (2018). Effect of uterosacral ligament suspension vs sacrospinous ligament fixation with or without perioperative behavioral therapy for pelvic organ vaginal prolapse on surgical outcomes and prolapse symptoms at 5 Years in the OPTIMAL randomized clinical trial. JAMA 319, 1554–1565. 10.1001/jama.2018.2827 29677302 PMC5933329

[B56] Jiang L. Xie C. Lung H. L. Lo K. W. Law G.-L. Mak N.-K. (2018). EBNA1-targeted inhibitors: novel approaches for the treatment of Epstein-Barr virus-associated cancers. Theranostics 8, 5307–5319. 10.7150/thno.26823 30555548 PMC6276081

[B57] Kalaria R. N. Maestre G. E. Arizaga R. Friedland R. P. Galasko D. Hall K. (2008). Alzheimer’s disease and vascular dementia in developing countries: prevalence, management, and risk factors. Lancet Neurology 7, 812–826. 10.1016/S1474-4422(08)70169-8 18667359 PMC2860610

[B58] Kang D.-W. Zhou S. Niranjan S. Rogers A. Shen C. (2024). Predicting operative time for metabolic and bariatric surgery using machine learning models: a retrospective observational study. Int. J. Surg. 110, 1968–1974. 10.1097/JS9.0000000000001107 38270635 PMC11019972

[B59] Karpuzoglu E. Schmiedt C. W. Pardo J. Hansen M. Guo T. L. Holladay S. D. (2014). Serine protease inhibition attenuates rIL-12-induced GZMA activity and proinflammatory events by modulating the Th2 profile from estrogen-treated mice. Endocrinology 155, 2909–2923. 10.1210/en.2014-1045 24840346 PMC4097994

[B60] Kim E. J. Chung N. Park S. H. Lee K.-H. Kim S. W. Kim J. Y. (2013). Involvement of oxidative stress and mitochondrial apoptosis in the pathogenesis of pelvic organ prolapse. J. Urology 189, 588–594. 10.1016/j.juro.2012.09.041 23260548

[B61] Kong Y.-L. Wang H.-D. Gao M. Rong S.-Z. Li X.-X. (2024). LncRNA XIST promotes bladder cancer progression by modulating miR-129-5p/TNFSF10 axis. Discov. Onc 15, 65. 10.1007/s12672-024-00910-8 PMC10917713 38446257

[B62] Kordzadeh A. Ramazani Saadatabadi A. Hadi A. (2020). Investigation on penetration of saffron components through lipid bilayer bound to spike protein of SARS-CoV-2 using steered molecular dynamics simulation. Heliyon 6, e05681. 10.1016/j.heliyon.2020.e05681 33344790 PMC7733551

[B63] Kuttan G. Pratheeshkumar P. Manu K. A. Kuttan R. (2011). Inhibition of tumor progression by naturally occurring terpenoids. Pharm. Biol. 49, 995–1007. 10.3109/13880209.2011.559476 21936626

[B64] Law S.-H. Chan M.-L. Marathe G. K. Parveen F. Chen C.-H. Ke L.-Y. (2019). An updated review of lysophosphatidylcholine metabolism in human diseases. IJMS 20, 1149. 10.3390/ijms20051149 30845751 PMC6429061

[B65] Le Stunff H. Véret J. Kassis N. Denom J. Meneyrol K. Paul J.-L. (2019). Deciphering the link between hyperhomocysteinemia and ceramide metabolism in alzheimer-type neurodegeneration. Front. Neurol. 10, 807. 10.3389/fneur.2019.00807 31417486 PMC6684947

[B66] Li B. S. Hong L. Min J. Wu D. B. Hu M. Guo W. J. (2013). The expression of glutathione peroxidase-1 and the anabolism of collagen regulation pathway transforming growth factor-beta1-connective tissue growth factor in women with uterine prolapse and the clinic significance. Clin. Exp. Obstet. Gynecol. 40, 586–590. 24597264

[B67] Li F. Qin W. Zhu M. Jia J. (2021a). Model-based projection of dementia prevalence in China and worldwide: 2020–2050. JAD 82, 1823–1831. 10.3233/JAD-210493 34219732

[B68] Li Q. Su X. Qing L. Xu W. Yang Y. You C. (2024). HER2 affects the biological behaviours of bladder cancer cells and is closely associated with the progression and prognosis of bladder cancer. Arch. Españoles Urol. 77, 79–91. 10.56434/j.arch.esp.urol.20247701.11 38374017

[B69] Li R. Qi J. Yang Y. Wu Y. Yin P. Zhou M. (2022). Disease burden and attributable risk factors of alzheimer’s disease and dementia in China from 1990 to 2019. J. Prev. Alzheimers Dis. 9, 306–314. 10.14283/jpad.2021.69 35543004

[B70] Li Y. Hong L. Liu C. Min J. Hong S. Hu M. (2017). Effect of puerarin on collagen metabolism of fibroblasts in pelvic tissue of women with pelvic organ prolapse. Mol. Med. Rep. 17, 2705–2711. 10.3892/mmr.2017.8112 29207080

[B71] Li Y. Nie N. Gong L. Bao F. An C. Cai H. (2021b). Structural, functional and molecular pathogenesis of pelvic organ prolapse in patient and Loxl1 deficient mice. Aging 13, 25886–25902. 10.18632/aging.203777 34923484 PMC8751609

[B72] Libeu C. P. Lund-Katz S. Phillips M. C. Wehrli S. Hernáiz M. J. Capila I. (2001). New insights into the heparan sulfate proteoglycan-binding activity of apolipoprotein E. J. Biol. Chem. 276, 39138–39144. 10.1074/jbc.M104746200 11500500

[B73] Lieberman J. (2010). Granzyme A activates another way to die. Immunol. Rev. 235, 93–104. 10.1111/j.0105-2896.2010.00902.x 20536557 PMC2905780

[B74] Lin C.-H. Yang H.-T. Lane H.-Y. D-glutamate (2019). D-glutamate, D-serine, and D-alanine differ in their roles in cognitive decline in patients with Alzheimer's disease or mild cognitive impairment. Pharmacol. Biochem. Behav. 185, 172760. 10.1016/j.pbb.2019.172760 31422081

[B75] Liu J.-H. Tsai T.-H. Chen Y.-J. Wang L.-Y. Liu H.-Y. Hsieh C.-H. (2020). Local irradiation modulates the pharmacokinetics of metabolites in 5-fluorouracil—radiotherapy–pharmacokinetics phenomenon. Front. Pharmacol. 11, 141. 10.3389/fphar.2020.00141 32174836 PMC7056828

[B76] Liu X. Wang F. Du W. Yang X. (2023). ESM-1 mediates cell progression in clear cell renal cell carcinoma by affecting wnt/β-catenin signalling pathway. Arch. Españoles Urol. 76, 290–297. 10.56434/j.arch.esp.urol.20237604.33 37455528

[B77] Lu L. Kang X. Yi B. Jiang C. Yan X. Chen B. (2022). Exploring the mechanism of yiqi qingre ziyin method in regulating neuropeptide expression for the treatment of atrophic rhinitis. Dis. Markers 2022, 4416637. 10.1155/2022/4416637 35299869 PMC8923799

[B78] Lye S. Aust C. E. Griffiths L. R. Fernandez F. (2021). Exploring new avenues for modifying course of progression of Alzheimer’s disease: the rise of natural medicine. J. Neurological Sci. 422, 117332. 10.1016/j.jns.2021.117332 33607542

[B79] Ma S. L. Tang N. L. S. Zhang Y. P. Ji L. Tam C. W. C. Lui V. W. C. (2008). Association of prostaglandin-endoperoxide synthase 2 (PTGS2) polymorphisms and Alzheimer’s disease in Chinese. Neurobiol. Aging 29, 856–860. 10.1016/j.neurobiolaging.2006.12.011 17234302

[B80] Malta T. M. Silva I. T. Pinheiro D. G. Santos A. R. D. Pinto M. T. Panepucci R. A. (2013). Altered expression of degranulation-related genes in CD8 ^+^ T cells in human T lymphotropic virus type I infection. AIDS Res. Hum. Retroviruses 29, 826–836. 10.1089/aid.2012.0205 23301858

[B81] Mattsson N. K. Karjalainen P. K. Tolppanen A.-M. Heikkinen A.-M. Sintonen H. Härkki P. (2020). Pelvic organ prolapse surgery and quality of life-a nationwide cohort study. Am. J. Obstet. Gynecol. 222, 588.e1–588.e10. 10.1016/j.ajog.2019.11.1285 31836546

[B82] Mazumder A. Cerella C. Diederich M. (2018). Natural scaffolds in anticancer therapy and precision medicine. Biotechnol. Adv. 36, 1563–1585. 10.1016/j.biotechadv.2018.04.009 29729870

[B83] Mei K. Chen Z. Wang Q. Ali A. Huang Y. Yi L. (2024). A prognostic aging-related lncRNA risk model correlates with the immune microenvironment in HCC. CI 3, 37–48. 10.58567/ci03020003

[B84] Michele S. Salluzzo M. G. Calogero A. E. Raffaele F. Bosco P. (2014). Association study of COX-2 (PTGS2) –765 G/C promoter polymorphism by pyrosequencing in Sicilian patients with Alzheimer’s disease. aoms 10 (6), 1235–1238. 10.5114/aoms.2014.47832 PMC4296078 25624863

[B85] Mohamed Yusof N. I. S. Mohd F. F. (2024). Nature’s toolbox for alzheimer’s disease: a review on the potential of natural products as alzheimer’s disease drugs. Neurochem. Int. 176, 105738. 10.1016/j.neuint.2024.105738 38616012

[B86] Nho K. Kueider-Paisley A. Arnold M. MahmoudianDehkordi S. Risacher S. L. Louie G. (2021). Serum metabolites associated with brain amyloid beta deposition, cognition and dementia progression. Brain Commun. 3, fcab139. 10.1093/braincomms/fcab139 34396103 PMC8361396

[B87] Nittayacharn P. Abenojar E. Cooley M. B. Berg F. M. Counil C. Sojahrood A. J. (2024). Efficient ultrasound-mediated drug delivery to orthotopic liver tumors – direct comparison of doxorubicin-loaded nanobubbles and microbubbles. J. Control. Release 367, 135–147. 10.1016/j.jconrel.2024.01.028 38237687 PMC11700473

[B88] Oinaka H. Kawakita F. Nakajima H. Suzuki Y. Nampei M. Okada T. (2024). Increased plasma periostin concentration predicts angiographic vasospasm development in non-severe aneurysmal subarachnoid hemorrhage. Brain Hemorrhages 5, 1–7. 10.1016/j.hest.2023.12.003

[B89] Pan P. Huang Y.-W. Oshima K. Yearsley M. Zhang J. Arnold M. (2019). The immunomodulatory potential of natural compounds in tumor-bearing mice and humans. Crit. Rev. Food Sci. Nutr. 59, 992–1007. 10.1080/10408398.2018.1537237 30795687 PMC6508979

[B90] Pang H. Zhang L. Han S. Li Z. Gong J. Liu Q. (2021). A nationwide population‐based survey on the prevalence and risk factors of symptomatic pelvic organ prolapse in adult women in China – a pelvic organ prolapse quantification system‐based study. BJOG Int. J. Obstet. Gy 128, 1313–1323. 10.1111/1471-0528.16675 PMC8252658 33619817

[B91] Paul B. D. Sbodio J. I. Snyder S. H. (2018). Cysteine metabolism in neuronal redox homeostasis. Trends Pharmacol. Sci. 39, 513–524. 10.1016/j.tips.2018.02.007 29530337 PMC5912966

[B92] Pesini A. Iglesias E. Bayona-Bafaluy M. P. Garrido-Pérez N. Meade P. Gaudó P. (2019). Brain pyrimidine nucleotide synthesis and Alzheimer disease. Aging 11, 8433–8462. 10.18632/aging.102328 31560653 PMC6814620

[B93] Piccialli I. Tedeschi V. Caputo L. D’Errico S. Ciccone R. De Feo V. (2022). Exploring the therapeutic potential of phytochemicals in alzheimer’s disease: focus on polyphenols and monoterpenes. Front. Pharmacol. 13, 876614. 10.3389/fphar.2022.876614 35600880 PMC9114803

[B94] Ravindranathan K. P. Mandiyan V. Ekkati A. R. Bae J. H. Schlessinger J. Jorgensen W. L. (2010). Discovery of novel fibroblast growth factor receptor 1 kinase inhibitors by structure-based virtual screening. J. Med. Chem. 53, 1662–1672. 10.1021/jm901386e 20121196 PMC2842983

[B95] Rempe R. G. Hartz A. M. Bauer B. (2016). Matrix metalloproteinases in the brain and blood–brain barrier: versatile breakers and makers. J. Cereb. Blood Flow. Metab. 36, 1481–1507. 10.1177/0271678X16655551 27323783 PMC5012524

[B96] Rijneveld A. W. Levi M. Florquin S. Speelman P. Carmeliet P. van der Poll T. (2002). Urokinase receptor is necessary for adequate host defense against pneumococcal pneumonia. J. Immunol. 168, 3507–3511. 10.4049/jimmunol.168.7.3507 11907112

[B97] Rogowski A. Bienkowski P. Tarwacki D. Dziech E. Samochowiec J. Jerzak M. (2015). Association between metabolic syndrome and pelvic organ prolapse severity. Int. Urogynecol J. 26, 563–568. 10.1007/s00192-014-2468-9 25047898 PMC4371825

[B98] Safarkhani M. Ojaghi A. Nezhad S. M. Daneshgar H. Paiva-Santos A. C. Radmanesh F. (2024). Engineered (NH2)-MIL-125(Ti)/copolymer@MnFe2O4 nanocomposite for synergistic eradication of cancer cells via DOX/pCRISPR delivery. Adv. Compos Hybrid. Mater 7, 18. 10.1007/s42114-023-00825-y

[B99] Seca A. Pinto D. (2018). Plant secondary metabolites as anticancer agents: successes in clinical trials and therapeutic application. IJMS 19, 263. 10.3390/ijms19010263 29337925 PMC5796209

[B100] Skorupski P. Jankiewicz K. Miotła P. Marczak M. Kulik-Rechberger B. Rechberger T. (2013). The polymorphisms of the MMP-1 and the MMP-3 genes and the risk of pelvic organ prolapse. Int. Urogynecol J. 24, 1033–1038. 10.1007/s00192-012-1970-1 23108733 PMC3671098

[B101] Smith E. R. Li Z. Chen Z.-S. Xu X.-X. (2023). Reassessing specificity/selectivity of taxane-based chemotherapy. Cancer Insight 3 (1), 37–48. 10.58567/ci03010002

[B102] Sonnaila S. Agrawal S. (2024). CRISPR-Cas9 unleashed: gene-slicing adventures in the cancer battlefield. Cancer Insight 2, 37–48. 10.58567/ci02020008

[B103] Strinic T. Vulic M. Tomic S. Capkun V. Stipic I. Alujevic I. (2009). Matrix metalloproteinases-1, -2 expression in uterosacral ligaments from women with pelvic organ prolapse. Maturitas 64, 132–135. 10.1016/j.maturitas.2009.08.008 19765922

[B104] Tanaka K. A. K. Kurihara S. Shibakusa T. Chiba Y. Mikami T. (2015). Cystine improves survival rates in a LPS-induced sepsis mouse model. Clin. Nutr. 34, 1159–1165. 10.1016/j.clnu.2014.11.014 25529480

[B105] Tanskanen M. Myllykangas L. Saarialho-Kere U. Paetau A. (2011). Matrix metalloproteinase-β19 expressed in cerebral amyloid angiopathy. Amyloid 18, 3–9. 10.3109/13506129.2010.541960 21261556

[B106] Tayeb B. A. Kusuma I. Y. Osman A. A. M. Minorics R. (2024). Herbal compounds as promising therapeutic agents in precision medicine strategies for cancer: a systematic review. J. Integr. Med. 22, 137–162. 10.1016/j.joim.2024.02.001 38462407

[B107] Te Braake F. W. J. Schierbeek H. Vermes A. Huijmans J. G. M. Van Goudoever J. B. (2009). High-dose cysteine administration does not increase synthesis of the antioxidant glutathione preterm infants. Pediatrics 124, e978–e984. 10.1542/peds.2008-2477 19822595

[B108] The Idiopathic Pulmonary Fibrosis Clinical Research Network Martinez F. J. de Andrade J. A. Anstrom K. J. King T. E. Jr Raghu G. (2014). Randomized trial of acetylcysteine in idiopathic pulmonary fibrosis. N. Engl. J. Med. 370, 2093–2101. 10.1056/NEJMoa1401739 24836309 PMC4116664

[B109] Thomas M. H. Pelleieux S. Vitale N. Olivier J. L. (2016). Dietary arachidonic acid as a risk factor for age-associated neurodegenerative diseases: potential mechanisms. Biochimie 130, 168–177. 10.1016/j.biochi.2016.07.013 27473185

[B110] Török N. Tanaka M. Vécsei L. (2020). Searching for peripheral biomarkers in neurodegenerative diseases: the tryptophan-kynurenine metabolic pathway. IJMS 21, 9338. 10.3390/ijms21249338 33302404 PMC7762583

[B111] Varnum M. M. Kiyota T. Ingraham K. L. Ikezu S. Ikezu T. (2015). The anti-inflammatory glycoprotein, CD200, restores neurogenesis and enhances amyloid phagocytosis in a mouse model of Alzheimer’s disease. Neurobiol. Aging 36, 2995–3007. 10.1016/j.neurobiolaging.2015.07.027 26315370 PMC4772879

[B112] Villaflores O. B. Chen Y.-J. Chen C.-P. Yeh J.-M. Wu T.-Y. (2012). Curcuminoids and resveratrol as anti-Alzheimer agents. Taiwan. J. Obstetrics Gynecol. 51, 515–525. 10.1016/j.tjog.2012.09.005 23276553

[B113] Wahi A. Bishnoi M. Raina N. Singh M. A. Verma P. Gupta P. K. (2024). Recent updates on nano-phyto-formulations based therapeutic intervention for cancer treatment. Oncol. Res. 32, 19–47. 10.32604/or.2023.042228 PMC10767243 38188681

[B114] Wang J. Wei R. Xie G. Arnold M. Kueider-Paisley A. Louie G. (2020). Peripheral serum metabolomic profiles inform central cognitive impairment. Sci. Rep. 10, 14059. 10.1038/s41598-020-70703-w 32820198 PMC7441317

[B115] Wang R. Reddy P. H. (2017). Role of glutamate and NMDA receptors in alzheimer’s disease. J. Alzheimers Dis. 57, 1041–1048. 10.3233/JAD-160763 27662322 PMC5791143

[B116] Wang Z. Zhao Z. Bai P. Ren J. Liu B. Naik N. (2024). The microstructure and property evolutions of Inconel718 lattice structure by selective laser melting. Adv. Compos Hybrid. Mater 7, 59. 10.1007/s42114-024-00869-8

[B117] Weintraub A. Y. Glinter H. Marcus-Braun N. (2020). Narrative review of the epidemiology, diagnosis and pathophysiology of pelvic organ prolapse. Int. braz J. Urol. 46, 5–14. 10.1590/s1677-5538.ibju.2018.0581 31851453 PMC6968909

[B118] William Raja T. R. Duraipandiyan V. Ignacimuthu S. Janakiraman U. Packiam S. M. (2023). Role of polyphenols in alleviating alzheimer’s disease: a review. CMC 30, 4032–4047. 10.2174/0929867330666221202152540 36476438

[B119] Withagen M. I. Milani A. L. Den Boon J. Vervest H. A. Vierhout M. E. (2011). Trocar-guided mesh compared with conventional vaginal repair in recurrent prolapse: a randomized controlled trial. Obstetrics & Gynecol. 117, 242–250. 10.1097/AOG.0b013e318203e6a5 21252735

[B120] Wu J.-J. Zhu S. Tang Y.-F. Gu F. Valencak T. G. Liu J.-X. (2023). Age- and microbiota-dependent cell stemness plasticity revealed by cattle cell landscape. Res. (Wash D C) 6, 0025. 10.34133/research.0025 PMC10076005 37040481

[B121] Xia S. Ma R. (2024). Tributyltin chloride induces chondrocyte damage through the activation of NLRP3-mediated inflammation and pyroptosis. Mol. Med. Rep. 30, 122. 10.3892/mmr.2024.13247 38785157 PMC11130746

[B122] Xia W. Jiang H. Tao E. Ye J. Wang F. Wang X. (2024). Comparison of ESIN and other minimally invasive techniques for anterior pelvic ring injury: a finite element analysis and case–control study. Int. J. Surg. 110, 2636–2648. 10.1097/JS9.0000000000001137 38320104 PMC11093452

[B123] Xiang Z. Xu M. Liao M. Jiang Y. Jiang Q. Feng R. (2015). Integrating genome-wide association study and brain expression data highlights cell adhesion molecules and purine metabolism in alzheimer’s disease. Mol. Neurobiol. 52, 514–521. 10.1007/s12035-014-8884-5 25204495

[B124] Xie J. Chen X. Zheng M. Zhu J. Mao H. (2024). The metabolism of coenzyme A and its derivatives plays a crucial role in diseases. Front. Biosci. Landmark Ed. 29, 143. 10.31083/j.fbl2904143 38682186

[B125] Yamada M. Ono K. Hamaguchi T. Noguchi-Shinohara M. (2015). “Natural phenolic compounds as therapeutic and preventive agents for cerebral amyloidosis,” in *Natural Compounds as therapeutic Agents for amyloidogenic diseases*. Advances in experimental medicine and biology. Editor Vassallo N. (Cham: Springer International Publishing), 79–94. 10.1007/978-3-319-18365-7_4 26092627

[B126] Yang H. Zonder J. A. Dou Q. P. (2009). Clinical development of novel proteasome inhibitors for cancer treatment. Expert Opin. Investigational Drugs 18, 957–971. 10.1517/13543780903002074 PMC3758888 19505187

[B127] Yang L. Xuan C. Yu C. Zheng P. Yan J. (2022). Diagnostic model of alzheimer’s disease in the elderly based on protein and metabolic biomarkers. JAD 85, 1163–1174. 10.3233/JAD-215119 34924381

[B128] Yao J.-Y. Yang Y.-L. Chen W.-J. Fan H.-Y. (2024). Exploring the therapeutic potential of Qi Teng Mai Ning recipe in ischemic stroke and vascular cognitive impairment. Tradit. Med. Res. 9, 57. 10.53388/TMR20240214001

[B129] Yin T. Mou S. Zhang H. Dong Y. Yan B. Huang W. (2024). CXCL10 could be a prognostic and immunological biomarker in bladder cancer. Discov. Onc 15, 148. 10.1007/s12672-024-00982-6 PMC11078901 38720149

[B130] Yu E. Liao Z. Fan W. Hu W. Tian G. Chen K. (2021). The economic burden of alzheimer’s disease in Zhejiang Province. JAD 80, 539–553. 10.3233/JAD-201285 33579844

[B131] Yue Y. Hsiao Y.-W. Zhou J.-B. (2020). Association between MMP/TIMP levels in the aqueous humor and plasma with axial lengths in myopia patients. BioMed Res. Int. 2020, 2961742. 10.1155/2020/2961742 32596291 PMC7305534

[B132] Zhang J.-F. Williams J. P. Zhao Q.-N. Liu H. An J.-X. (2023). Combined high-voltage pulsed radiofrequency and ozone therapy versus ozone therapy alone in treating postherpetic neuralgia: a retrospective comparison. Med. Gas. Res. 13, 15–22. 10.4103/2045-9912.352660 35946218 PMC9480353

[B133] Zhang Y. Lin N. Liu X. Yao T. (2024). Dishevelled segment polarity protein 3 (DVL3) induced by bacterial LPS promotes the proliferation and migration of prostate cancer cells through the TLR4 pathway. Arch. Españoles Urol. 77, 193–201. 10.56434/j.arch.esp.urol.20247702.25 38583012

[B134] Zhang Z. Li Q. Wang F. Ma B. Meng Y. Zhang Q. (2021). Identifying hypoxia characteristics to stratify prognosis and assess the tumor immune microenvironment in renal cell carcinoma. Front. Genet. 12, 606816. 10.3389/fgene.2021.606816 34194463 PMC8238406

[B135] Zhao X. Ma C. Li R. Xue J. Liu L. Liu P. (2017). Hypoxia induces apoptosis through HIF-1 *α* signaling pathway in human uterosacral ligaments of pelvic organ prolapse. BioMed Res. Int. 2017, 8316094–8316098. 10.1155/2017/8316094 29230415 PMC5688353

[B136] Zhao Y. Chen H. Iqbal J. Liu X. Zhang H. Xiao S. (2021a). Targeted metabolomics study of early pathological features in hippocampus of triple transgenic Alzheimer’s disease male mice. J. Neurosci. Res. 99, 927–946. 10.1002/jnr.24750 33197957

[B137] Zhao Y. Li M. Yang Y. Wu T. Huang Q. Wu Q. (2021b). Identification of macrophage polarization-related genes as biomarkers of chronic obstructive pulmonary disease based on bioinformatics analyses. BioMed Res. Int. 2021, 9921012–9921017. 10.1155/2021/9921012 34250093 PMC8238569

[B138] Zhao Y. Xia Z. Lin T. Yin Y. (2020). Significance of hub genes and immune cell infiltration identified by bioinformatics analysis in pelvic organ prolapse. PeerJ 8, e9773. 10.7717/peerj.9773 32874785 PMC7441923

[B139] Zhou Q. Hong L. Wang J. (2018). Identification of key genes and pathways in pelvic organ prolapse based on gene expression profiling by bioinformatics analysis. Arch. Gynecol. Obstet. 297, 1323–1332. 10.1007/s00404-018-4745-1 29546564

[B140] Zong W. Meyn L. A. Moalli P. A. (2009). The amount and activity of active matrix metalloproteinase 13 is suppressed by estradiol and progesterone in human pelvic floor fibroblasts. Biol. Reproduction 80, 367–374. 10.1095/biolreprod.108.072462 PMC2804822 18987329

[B141] Zong W. Stein S. E. Starcher B. Meyn L. A. Moalli P. A. (2010). Alteration of vaginal elastin metabolism in women with pelvic organ prolapse. Obstetrics & Gynecol. 115, 953–961. 10.1097/AOG.0b013e3181da7946 PMC3042770 20410768

