# Supplementary material for: The role of GZMA as a target of cysteine and biomarker in Alzheimer’s disease, pelvic organ prolapse, and tumor progression
Source: Front Pharmacol. 2024 Aug 20;15:1447605. doi: 10.3389/fphar.2024.1447605 (PMC11368878; doi:10.3389/fphar.2024.1447605)
Supplement: Supplementary file 1 [file DataSheet1.docx]

**Supplementary Figures**


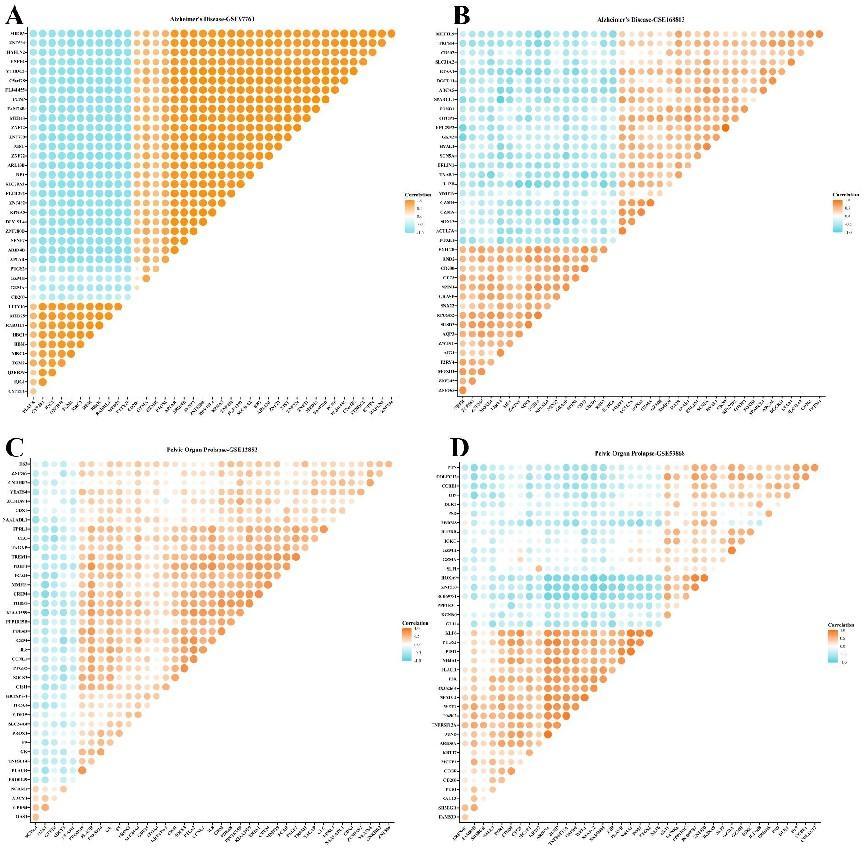


**Supplementary Figure 1. Differential gene expression evaluation throughout numerous Gene Expression Omnibus (GEO) datasets associated with ailment research.**

(A) Reciprocal map of differentially expressed genes (Dogs) inside the AD dataset GSE97760. This heatmap represents the correlation among Dogs recognized within the GSE97760 dataset. The graduation scale shows the diploma of correlation, with orange representing fantastic correlations and blue representing poor correlations.

(B) Reciprocal map of Dogs inside the dataset GSE168813, any other observation associated with AD. This plot suggests the correlation amongst Dogs with inside the GSE168813 dataset, with graduation coding much like panel A. The dataset affords perception of the gene expression changes in Alzheimer's Disease.

(C) Interaction map of Dogs in GSE12852, detailing the interactions among Dogs. This parent highlights the interplay styles of genes differentially expressed inside the GSE12852 dataset and the usage of the equal graduation scale for correlation.

(D) Reciprocal plot of Dogs with inside the POP dataset GSE53868. This plot offers the correlation amongst Dogs recognized with inside the GSE53868 dataset, which researches gene expression modifications associated with postoperative cognitive dysfunction.


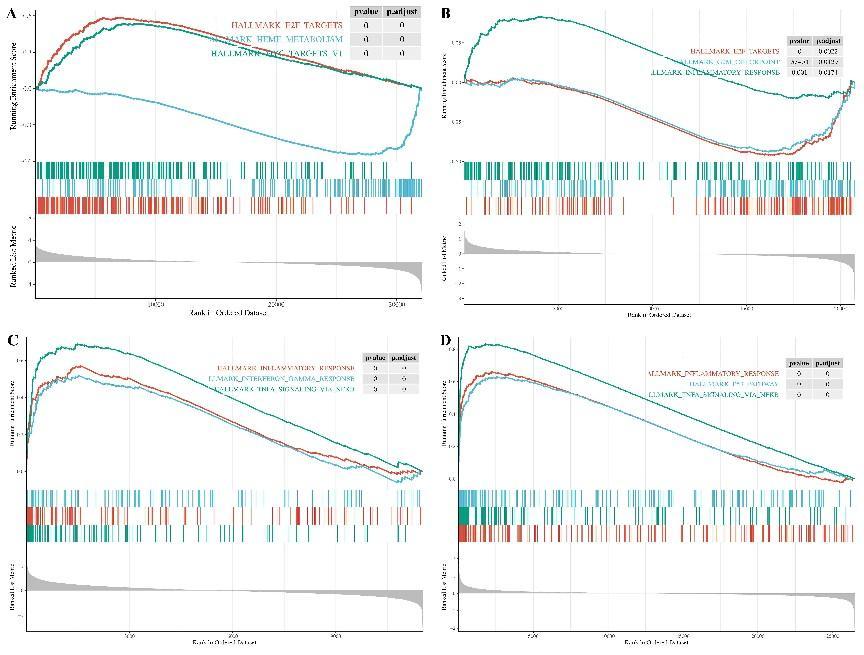


**Supplementary Figure 2. GSEA of a couple of gene expression datasets to pick out enriched organic strategies and pathways.**

(A) GSEA of AD dataset GSE97760: This plot depicts the enrichment score (ES) across the ranked list of genes. The strolling ES for gene sets described through previous organic expertise is shown, with the peak height indicating the point at which the gene sets are most significantly overrepresented on the pinnacle or backside of the ranked listing. The enrichment plot includes the ES curve, the positions of gene sets, and the strolling ES.

(B) GSEA for the AD dataset GSE168813: Similar layout to (A), illustrating the ES curve and bars representing gene positions. The plot suggests the enrichment of particular gene sets within the ranked list of genes from the AD dataset.

(C) GSEA of dataset GSE12852: This dataset includes an extraordinary neurological condition. The plot shows the running ES, positions of gene sets, and peak ES score, indicating the enrichment of biological processes relevant to the condition studied.

(D) GSEA of the dataset GSE53868: The plot shows the ES and the distribution of gene sets across the ranked list of genes, is consistent with the other panels. Focusing on POP, this dataset reveals the enrichment of gene sets associated with this condition.


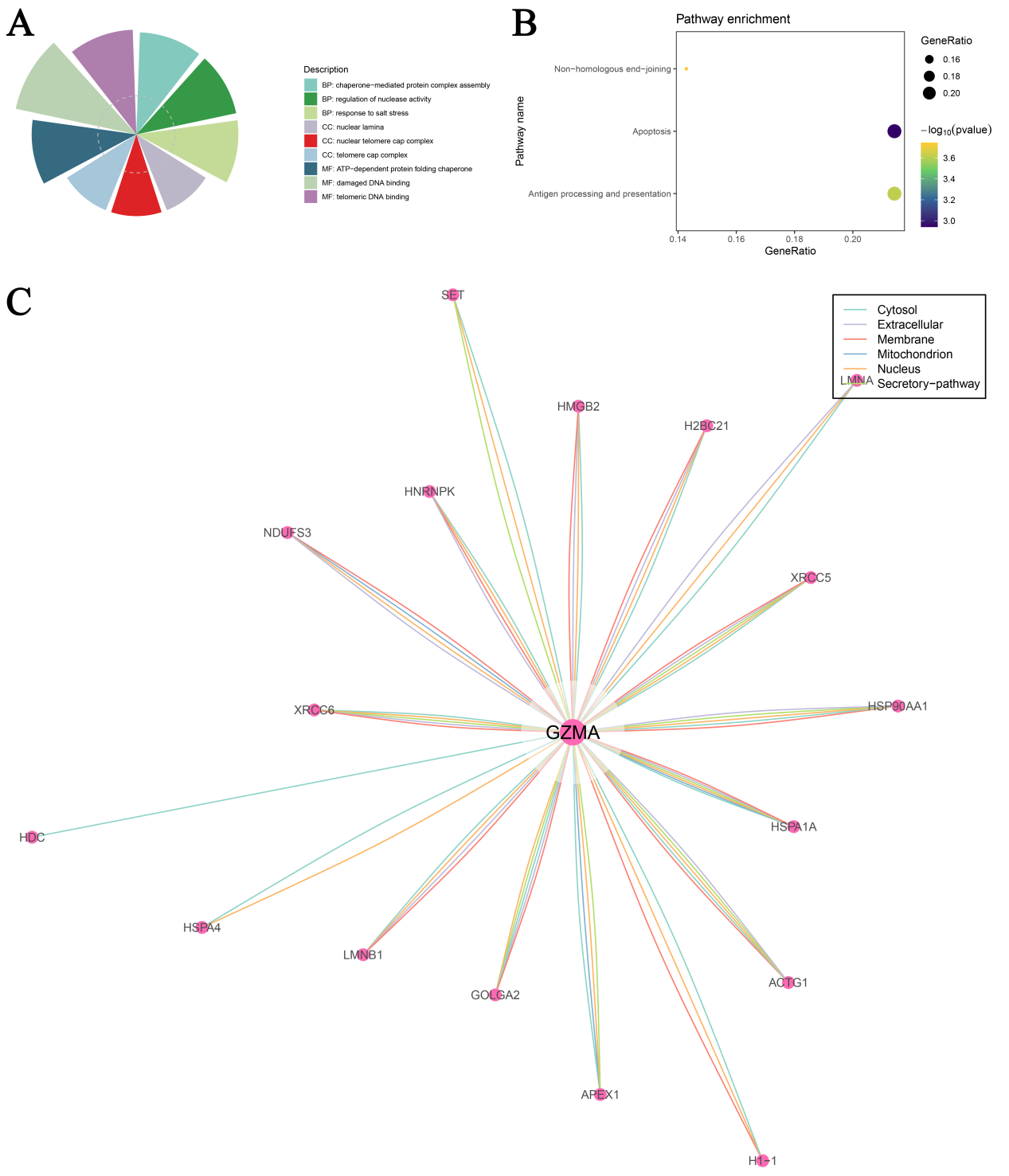


**Supplementary Figure 3. GZMA Single Gene Enrichment Analysis**

(A) GZMA Interaction PPI Network: The interaction network for GZMA was generated using the STRING database. This network encompasses a range of interacting proteins, differentiated by their subcellular localization: cytosol, extracellular, membrane, mitochondrion, nucleus, and secretory pathway. The depicted connections highlight both known and predicted protein-protein interactions involving GZMA.

(B) GO/KEGG Enrichment Analysis: Pathway enrichment analysis for GZMA was conducted utilizing the Gene Ontology (GO) and Kyoto Encyclopedia of Genes and Genomes (KEGG) databases. The top pathways are represented by the size and color intensity of the dots, where larger dots signify higher gene ratios and darker colors represent more significant p-values. Notable pathways include apoptosis, non-homologous end joining, and antigen processing and presentation.

(C) GO/KEGG Enrichment Network: This network diagram illustrates the significant GO and KEGG terms associated with GZMA. Each line signifies a connection to a pertinent biological process, cellular component, or molecular function. This network aids in elucidating the intricate relationships and pathways in which GZMA plays a role.
